# Combining genetic and demographic information to prioritize conservation efforts for anadromous alewife and blueback herring

**DOI:** 10.1111/eva.12111

**Published:** 2013-10-02

**Authors:** Eric P Palkovacs, Daniel J Hasselman, Emily E Argo, Stephen R Gephard, Karin E Limburg, David M Post, Thomas F Schultz, Theodore V Willis

**Affiliations:** 1 Department of Ecology and Evolutionary Biology, University of CaliforniaSanta Cruz, CA, USA; 2 Inland Fisheries Division, Connecticut Department of Energy and Environmental ProtectionOld Lyme, CT, USA; 3 Department of Environmental and Forest Biology, College of Environmental Science and Forestry, State University of New YorkSyracuse, NY, USA; 4 Department of Ecology and Evolutionary Biology, Yale UniversityNew Haven, CT, USA; 5 Division of Marine Science and Conservation Nicholas School of the Environment, Duke UniversityBeaufort, NC, USA; 6 Department of Environmental Science, University of Southern MaineGorham, ME, USA

**Keywords:** demography, distinct population segments, ecological restoration, microsatellites, population genetics, population trends, stock structure, time series

## Abstract

A major challenge in conservation biology is the need to broadly prioritize conservation efforts when demographic data are limited. One method to address this challenge is to use population genetic data to define groups of populations linked by migration and then use demographic information from monitored populations to draw inferences about the status of unmonitored populations within those groups. We applied this method to anadromous alewife (*Alosa pseudoharengus*) and blueback herring (*Alosa aestivalis*), species for which long-term demographic data are limited. Recent decades have seen dramatic declines in these species, which are an important ecological component of coastal ecosystems and once represented an important fishery resource. Results show that most populations comprise genetically distinguishable units, which are nested geographically within genetically distinct clusters or stocks. We identified three distinct stocks in alewife and four stocks in blueback herring. Analysis of available time series data for spawning adult abundance and body size indicate declines across the US ranges of both species, with the most severe declines having occurred for populations belonging to the Southern New England and the Mid-Atlantic Stocks. While all alewife and blueback herring populations deserve conservation attention, those belonging to these genetic stocks warrant the highest conservation prioritization.

## Introduction

The inherent value of integrating genetic and demographic data in the design of conservation and recovery plans has been recognized for some time, particularly in the context of evaluating extinction risk in small, isolated populations (Lande 1988; Jamieson and Allendorf 2012). A somewhat different perspective that has received less attention is the combination of genetic and demographic information to define management units and prioritize populations within those units for conservation action (Wood and Gross 2008). This approach recognizes that population genetic structure is the outcome of demographic nonindependence caused by migration (Waples and Gaggiotti 2006). The complementarity of genetic and demographic data may be especially useful when demographic data are limited, yet broad conservation prioritization is required. In this circumstance, population genetic data can be used to define demographically linked groups of populations (e.g., clusters or stocks), and then, demographic information from a subset of populations can be used to draw inferences about the status of other populations within those groups. This approach allows both monitored and unmonitored populations to be included in conservation prioritizations, which is critical for the management of species for which long-term demographic data are limited to just a few populations.

Here, we apply this framework to define management units and prioritize conservation actions for anadromous alewife (*Alosa pseudoharengus*) and blueback herring (*Alosa aestivalis*) – species for which demographic information is limited to a handful of rivers Atlantic States Marine Fisheries Commission (ASMFC 2012). River herring (as the species are collectively known) are native to the Atlantic Coast of North America. Historically, blueback herring ranged from the southern Gulf of St. Lawrence to the St. Johns River, Florida and alewife ranged from Labrador to South Carolina (Loesch 1987). These species represent an important ecological component of coastal marine and freshwater ecosystems. They are keystone species in coastal lakes (Post et al. 2008), an important agent of nutrient transport between marine and freshwater food webs (West et al. 2010), and a prey resource for coastal birds and fishes (Walter and Austin 2003; Jones et al. 2010). The local ecological benefits derived from anadromous alewife and blueback herring depend on abundant spawning runs throughout their ranges.

The fishery for alewife and blueback herring is one of the oldest in North America. Population declines became pronounced as early as the mid-1700s and included overall reductions in abundance (Hall et al. 2012) as well as the loss of unique spawning forms (or morphs) that may have represented genetically distinct subpopulations (Chapman 1884). Early declines were likely the result of overharvest, dam construction, and reduced water quality (Hightower et al. 1996; Limburg and Waldman 2009; Hall et al. 2011, 2012). Despite early declines, US coastwide fisheries landings remained stable from 1950–1969 (ASMFC 2012). Starting in 1970, landings declined sharply and have since fallen by 93% (ASMFC 2012). In addition, there is evidence for harvest-induced changes in life history traits (Davis and Schultz 2009), climate-induced shifts in migration timing (Ellis and Vokoun 2009), and an ongoing southern range contraction in alewife that has resulted in population extirpations from South Carolina and possibly southern North Carolina (E. P. Palkovacs, T. F. Schultz and A. S. Overton, unpublished data).

The rate and magnitude of the decline in commercial river herring landings is on par with well-publicized declines of Atlantic cod (*Gadus morhua*) (Mayo and Col 2006; O'Brien et al. 2006). However, river herring declines were largely overlooked until recently. Between 2005 and 2007, alewife and blueback herring were declared Species of Concern by the National Marine Fisheries Service (NMFS), and harvest restrictions were put in place in Massachusetts, Rhode Island, Connecticut, and North Carolina. Starting in 2012, harvest restrictions were extended to all coastal states. The ecological and cultural importance of alewife and blueback herring and the magnitude of recent declines make clear the need for conservation action, but how to designate management units and prioritize recovery efforts across those units has been equivocal. For example, Distinct Population Segments proposed in a recent Endangered Species Act petition [NRDC (Natural Resources Defense Council) 2011] were based on regional differences in habitat, climate, and geology but included no biological justifications based on population genetic structure or other characteristics of populations. By assessing population genetic structure at multiple spatial scales, and associating that structure with recent demographic trends in spawning adult abundance (run size) and body size (mean length), we provide important information to designate management units and to prioritize populations within those units for restoration efforts.

## Materials and methods

### Study system

Alewife and blueback herring belong to the family Clupeidae. Their predominant life history form is anadromy, although both species can form freshwater resident populations. Mature adults migrate from the ocean into coastal streams and rivers in the spring to spawn. The onset of spawning begins about 3–4 weeks earlier in the year for alewife than for blueback herring (Loesch 1987). Juveniles typically rear in freshwater for several months before migrating to the ocean to mature at between 3 and 6 years of age. Both species are iteroparous, although decreased rates of repeat-spawning have been observed for some populations (Davis and Schultz 2009; ASMFC 2012).

### Genetic analysis

#### Sample collections

**Figure 1:**
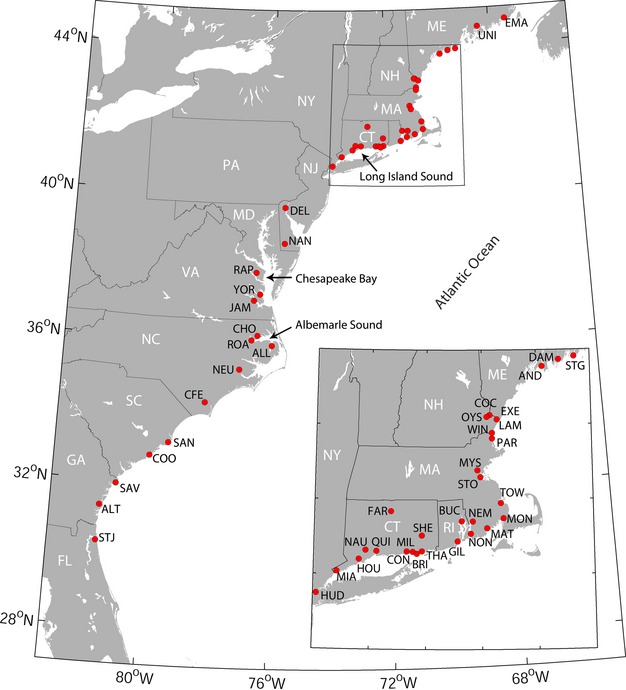
Coastal rivers in Eastern North America examined in this study spanned the US range of alewife and blueback herring. Sites indicated on the map include rivers sampled for genetic analysis and rivers included in the analysis of demographic time series data. River names and datasets associated with each sample code are provided in Table 1.

We sampled across the US range of anadromous alewife and blueback herring from 2008–2012 (Fig. 1) and targeted 50 specimens per collection. Sampling effort provided muscle or fin tissue from 947 alewife and 1183 blueback herring from 20 spawning rivers per species (Table 1). Tissue samples were obtained from adult and juvenile specimens captured on or near their freshwater spawning grounds and preserved in 95% ethanol until DNA extraction.

**Table 1:** Datasets included in population genetic and demographic analyses

				Microsatellites		Demographic time series		
	River	Code	State	Sample year(s)	*N*	Mean length	Run size (Counts)	Run size (CPUE)
**Alewife**								
1	East Machias	EMA	ME	2010	58			
2	Union	UNI	ME				1982–2010	
3	St George	STG	ME	2010	69			
4	Damariscotta	DAM	ME				1977–2010	
5	Androscoggin	AND	ME			1986–2010	1983–2010	
6	Coheco	COC	NH			1992–2010		
7	Exeter	EXE	NH			1992–2010		
8	Lamprey	LAM	NH	2010	47	1990–2010		
9	Winnicut	WIN	NH			1998–2009		
10	Parker	PAR	MA				1972–78, 1997–2010	
11	Mystic	MYS	MA	2010	68			
12	Stony Brook	STO	MA			1979–2004		
13	Town Brook	TOW	MA	2011	46			
14	Monument	MON	MA	2011	49	1984–2010	1980–2010	
15	Mattipoisett	MAT	MA				1988–2010	
16	Nemasket	NEM	MA				1996–2010	
17	Nonquit	NON	RI				1999–2010	
18	Buckeye Brook	BUC	RI				2003–2010	
19	Gilbert Stuart	GIL	RI	2011	44		1981–2010	
20	Thames	THA	CT	2009	36			
21	Shetucket	SHE	CT				2003–2010	
22	Bride Brook	BRI	CT	2009	34		2003–2010	
23	Mill Brook	MIL	CT				2002–2010	
24	Connecticut	CON	CT	2009, 2011	7, 26			
25	Farmington	FAR	CT				2003–2010	
26	Quinnipiac	QUI	CT	2009	25			
27	Naugatuck	NAU	CT				2003–2006	
28	Housatonic	HOU	CT	2008, 2009	13, 25			
29	Mianus	MIA	CT	2009	25		2005–2010	
30	Hudson	HUD	NY	2009, 2012	13, 48	1980–2010		
31	Delaware	DEL	NJ	2011	42			
32	Nanticoke	NAN	MD	2011	58	1991–2007		
33	Rappahannock	RAP	VA	2011	62			1994–2010
34	York	YOR	VA					1994–2010
35	James	JAM	VA					1994–2010
36	Chowan	CHO	NC	2011	54	1972–2009	1972–2003	1977–2006
37	Roanoke	ROA	NC	2011	49			
38	Alligator	ALL	NC	2011	49			
**Blueback herring**								
1	East Machias	EMA	ME	2010	57			
2	St George	STG	ME	2010	42			
3	Exeter	EXE	NH	2010	41			
4	Cocheco	COC	NH			1992–2008		
5	Oyster	OYS	NH			1992–2010		
6	Winnicut	WIN	NH			1998–2009		
7	Mystic	MYS	MA	2010	66			
8	Monument	MON	MA	2011	50	1984–2010	1980–2010	
9	Gilbert Stuart	GIL	RI	2011	38			
10	Shetucket	SHE	CT				2003–2010	
11	Connecticut	CON	CT	2008, 2009, 2011	34, 62, 46		1966–2011	
12	Farmington	FAR	CT				2003–2010	
13	Naugatuck	NAU	CT				2003–2010	
14	Mianus	MIA	CT				2005–2010	
15	Hudson	HUD	NY	2009	77	1976–2010		
16	Delaware	DEL	NJ	2011	48			
17	Nanticoke	NAN	MD	2011	24	1989–2007		
18	Rappahannock	RAP	VA	2011	58			
19	James	JAM	VA	2011	97			
20	Chowan	CHO	NC	2010, 2011	12, 58	1972–2009	1972–2009	1977–2006
21	Roanoke	ROA	NC	2011	50			
22	Neuse	NEU	NC	2011	65			
23	Cape Fear	CFE	NC	2011	57			
24	Santee	SAN	SC	2011	61	1991–2010	1980–1990	1990–2010
25	Cooper	COO	SC					1969–2008
26	Savannah	SAV	GA	2011	51			
27	Altamaha	ALT	GA	2011	52			
28	St Johns	STJ	FL	2011	37	1972–73, 2001–07		

For genetic analyses, the collection year(s) and sample sizes per year (*N*) are given. For demographic time series, the years spanning each time series are indicated.

#### Laboratory protocols

Genomic DNA was extracted from tissues using one of two methods: Promega Wizard® SV Genomic DNA Purification System or 10% Chelex 100 (Bio-Rad, Richmond, CA). Genomic DNA was stored at −20° C. Specimens were genotyped at 15 polymorphic microsatellite loci (*Aa046, Aa070, Aa074, Aa081, Aa082, Aa091, Aa093, Ap010, Ap033, Ap037, Ap038, Ap047, Ap058, Ap070, Ap071*). Amplification, size-fragment analysis, and scoring were conducted following A'Hara et al. (2012). To confirm consistency in scoring and reproducibility of genotypes, positive and negative controls were used.

### Population genetic analysis

#### Data conformance to model assumptions

Genotyping artifacts were assessed using MICROCHECKER v.2.2.3 (Van Oosterhout et al. 2004). Tests for departures from Hardy–Weinberg equilibrium (HWE) and linkage disequilibrium (LD) were performed with GENEPOP v.4.0.6 (Rousset 2008) using default parameters for all tests. Sequential Bonferroni adjustments were used to judge significance levels for all simultaneous tests (Holm 1979; Rice 1989). Selective neutrality of the microsatellite markers used in this study was evaluated using relative variance in repeat number (lnRV) and heterozygosity (lnRH) (Schlotterer 2002; Schlotterer and Deiringer 2005).

#### Genetic diversity

For each river, the number of alleles per locus (*N*_a_), observed heterozygosity (*H*_O_), an unbiased estimate of expected heterozygosity (*H*_E_) (Nei 1978), and inbreeding coefficient (*F*_IS_) (Weir and Cockerham 1984) were calculated using GENETIX v.4.05 (Belkhir et al. 2004). Allelic richness (*R*) per locus was calculated for each river using FSTAT 2.9.3.2 (Goudet 2001) standardized to a minimum sample size of 24 individuals for alewife, and 26 individuals for blueback herring (Leberg 2002).

#### Genetic differentiation

The statistical power and realized α-error for testing the null hypothesis of genetic homogeneity among rivers was assessed using POWSIM (Ryman and Palm 2006). Allelic heterogeneity among rivers was assessed via genic tests in GENEPOP v.4.0.6 (Rousset 2008) using default parameters for all tests. Tests were combined across loci or collections using Fisher's method. Hierarchical amova was conducted to partition components of genetic variation among rivers, among collections, and among individuals within collections, using a permutation procedure (10 000 iterations) in Arlequin 3.1 (Excoffier 2005).

Overall and pairwise *F*_ST_ values (θ) (Weir and Cockerham 1984) were estimated using FSTAT (Goudet 2001). The effect of variation in genetic diversity on genetic differentiation (Hedrick 2005) was accounted for by calculating standardized estimates of differentiation (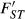) using RECODEDATA v.0.1 (Meirmans 2006) together with FSTAT to estimate *F*_ST(max)_ for each pairwise comparison. Standardized estimates of differentiation were then calculated as 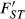
 = *F*_ST_/*F*_ST(max)_ (Hedrick 2005).

#### Relationships among populations

Genetic affinities among rivers were examined using principal coordinates analysis (PCoA) of the pairwise genetic distance matrix for *D*_A_ (Nei et al. 1983) implemented in GenAlEx v.6.0 (Peakall and Smouse 2006).

#### Population structure

Two Bayesian model-based clustering methods, implemented in STRUCTURE v.2.3.3 (Pritchard et al. 2000; Falush et al. 2003) and BAPS v.5.3 (Corander et al. 2006), respectively, were used concomitantly in a hierarchical approach to infer the number of genetically homogenous clusters among rivers (Latch et al. 2006). For STRUCTURE, a burn-in of 50 000 replicates was followed by 250 000 replicates of the Markov Chain Monte Carlo (MCMC) simulation, employing the admixture model and correlated allele frequencies among populations. Three iterations of this parameter set were performed for *K* (number of clusters) from 1 to 13, allowing an estimation of the most likely number of clusters. Both the plateau of likelihood values (Pritchard et al. 2000) and Δ*K* (i.e., second order rate of change between successive *K* values) (Evanno et al. 2005) were estimated.

For BAPS, the mixture model was first applied to cluster groups of individuals based on their multilocus genotypes. Three iterations of *K* (1–13) were conducted among populations to determine the number of genetically homogeneous groups. Admixture analysis was then conducted to estimate individual admixture proportions with regards to the most likely number of *K* clusters identified (Corander and Marttinen 2006), and visualized using DISTRUCT v.1.1 (Rosenberg 2004). Results from STRUCTURE and BAPS were used to delineate stocks for the purpose of examining stock-specific demographic trends in mean length of spawning adults and spawning adult run size.

#### Isolation by distance

Analysis of isolation by distance (IBD) was conducted among rivers to test for correlations between geographic distance and genetic differentiation using 10 000 permutations of the Mantel test implemented in IBDWS v.3.15 (Jensen et al. 2005). Pairwise 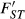 values were linearized (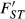/(1−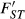)) following Rousset (2008). Geographic distance between river mouths was measured using the Gebco 1-min global bathymetry grid to identify land and ocean pixels. A Multistencil Fast Marching Method algorithm implemented in MATLAB (MathWorks, Natick, MA) was then used to find the distances from each river mouth to each other pixel on the globe. The shortest path distance between river mouths was then calculated by summing the Euler distances for each pixel step and converting from degrees to kilometers.

### Demographic analysis

#### Data collection

We obtained demographic time series data from the ASMFC River Herring Benchmark Stock Assessment (hereafter Stock Assessment; ASMFC 2012). For alewife, we analyzed demographic time series from 27 rivers from Maine to North Carolina (Table 1). For blueback herring, we analyzed time series from 15 rivers from Maine to Florida (Table 1). For demographic variables, we examined the mean total length of spawning adults and spawning adult run size. Other demographic variables involving age estimates (maximum age, length-at-age, age-at-maturity) were reported in the Stock Assessment but are not analyzed here because inconsistencies in aging techniques were deemed to make age data unreliable (ASMFC 2012). For mean length, data were collected for females and males separately, with one exception (Stony Brook, Massachusetts alewife). For run size estimates, data were based either on adult run counts (for fisheries-independent data) or measures of catch-per-unit effort (CPUE; for fisheries-dependent data). Run size data were normalized [(observed−mean)/standard deviation] as reported in the Stock Assessment (ASMFC 2012).

### Time series analysis

#### Demographic trends by time series

For each time series, we estimated the nonparametric linear regression slope (Theil-Sen slope) and tested for significant trends over time using Mann–Kendall tests. Both procedures were conducted using Package ‘rkt’ (Marchetto 2012) implemented in R (R Development Core Team 2011). We examined trends for each time series independently across all years sampled.

#### Demographic trends by species and stock

We used general linear models to test for differences in demographic trends between species and among stocks within each species. Many populations for which we had time series information were also included in our genetic analysis, making stock assignments unambiguous (Table 1). Populations not sampled for genetics were assigned to stocks based on geographic proximity to sampled rivers. The nonparametric linear regression slope (hereafter slope) of each time series was used as the dependent variable. We conducted analyses using slope values estimated from each time series, with ‘species’ or ‘stock’ included as fixed factors in the model. For among-stock comparisons of mean length, we also included ‘sex’ in the model as a fixed factor. We used *post hoc* Tukey's HSD tests to examine pairwise differences between stocks. General linear models and *post hoc* tests were conducted using PASW Statistics 18.0 (IBM Corporation, Somers, NY).

#### Conservation prioritization

We combined genetic and demographic data to develop a quantitative conservation prioritization for river herring populations that the Stock Assessment identified as being of current or historical importance. We examined the distribution of slope values for mean length and run size time series (both species examined together). We considered demographically increasing populations (slope > 0) to be low priority (i.e., at low risk), stable or slightly declining populations as medium priority, and steeply declining populations as high priority. We set the thresholds between medium and high priority populations at slope = −0.75 for mean length and slope = −0.05 for run size. These values resulted in approximately equal numbers of cases being categorized as medium and high priority. In cases where mean length and run size data were both available but designations did not agree (e.g., mean length gave a prioritization of ‘medium’ and run size gave a prioritization of ‘high’), we applied the more conservative designation (e.g., in this case ‘high’) due to the precautionary principle. We used genetic information to extend conservation prioritization to demographically unmonitored populations. We assigned all populations to genetic stocks as described above and calculated the average slope values for each genetic stock. These average slope values were used to designate stock-level prioritizations, which were then applied to any unmonitored rivers within a given stock.

## Results

### Genetic analysis

#### Data conformance to model assumptions

Evidence for null alleles resulted in the exclusion of loci for both alewife (*Aa082*, *Ap037, Ap047, Ap070*) and blueback herring (*Aa081, Ap058*) prior to further analyses. Remaining loci were retained as evidence for null alleles was sporadically distributed among loci and rivers. Exact tests revealed that genotypic frequencies were largely in accordance with HWE for both species (*P* > 0.05; sequential Bonferroni correction for 20 comparisons). HWE departures for alewife and blueback herring remained for 11 and 20 locus river comparisons, respectively, and were due to heterozygote deficiencies from sporadic null alleles. Exact tests of LD revealed that loci were physically unlinked and statistically independent (*P* > 0.05; sequential Bonferroni correction for 1100 and 1560 comparisons for alewife and blueback herring, respectively). Relative variance in repeat number (lnRV) and heterozygosity (lnRH) failed to detect outlier loci for either species, and provided no evidence of non-neutrality.

#### Genetic diversity

Genetic polymorphism varied for both alewife and blueback herring depending on the locus and river considered (Tables S1 and S2). For alewife, the number of alleles per locus ranged from 5 (*Aa046*) to 19 (*Ap010*). *H*_o_ varied from 0.50 (Town Brook) to 0.67 (Delaware), and *R* from 4.00 (Lamprey) to 5.49 (Delaware) (Table S1). For blueback herring, the number of alleles per locus ranged from 7 (*Ap047, Aa091*) to 28 (*Ap037*). *H*_O_ varied from 0.50 (Gilbert Stuart) to 0.57 (Nanticoke), and *R* from 4.59 (Monument) to 6.81 (Delaware) (Table S2).

#### Genetic differentiation

An assessment of statistical power indicated that our microsatellite loci provided sufficient resolution to detect weak differentiation among alewife and blueback herring populations. The probability of obtaining a significant (*P* < 0.05) result in contingency tests among populations with an *F*_ST_ of 0.001 was 0.86 and 0.98 (χ^2^) for alewife and blueback herring, respectively, while maintaining the realized α-error at the intended level (0.05) for tests of genetic homogeneity.

For alewife, significant (*P* < 0.05) genic differentiation between populations was observed for 179/190 pairwise comparisons, with nonsignificant comparisons occurring among neighboring and geographically proximal populations (Table 2). For blueback herring, significant (*P* < 0.05) genic differentiation between populations was observed for 178/190 pairwise comparisons, with nonsignificant comparisons occurring predominately among neighboring and geographically proximal rivers in the center of the species range (Table 3).

**Table 2:** Probability values for pairwise tests of genic heterogeneity among alewife populations

	EMA	STG	LAM	MYS	MON	TOW	GIL	THA	BRI	CON	QUI	HOU	MIA	HUD	DEL	NAN	RAP	CHO	ROA
STG	0.000																		
LAM	0.000	**0.585**																	
MYS	0.000	0.000	0.000																
MON	0.000	0.000	0.000	0.000															
TOW	0.000	0.000	0.000	0.000	0.000														
GIL	0.000	0.000	0.000	0.000	0.002	0.000													
THA	0.000	0.000	0.000	0.000	0.000	0.000	0.026												
BRID	0.000	0.000	0.000	0.000	0.000	0.000	0.000	**0.208**											
CON	0.000	0.000	0.000	0.000	0.000	0.000	0.000	0.024	0.000										
QUI	0.000	0.000	0.000	0.000	0.000	0.000	0.003	**0.512**	**0.070**	0.003									
HOU	0.000	0.000	0.000	0.020	0.000	0.000	0.000	**0.176**	0.002	0.001	**0.089**								
MIA	0.000	0.000	0.000	0.000	0.000	0.000	0.000	0.000	0.000	0.000	0.000	0.000							
HUD	0.000	0.000	0.000	0.000	0.000	0.000	0.000	0.012	0.000	0.000	0.014	**0.062**	0.000						
DEL	0.000	0.000	0.000	0.000	0.000	0.000	0.000	0.001	0.000	0.000	0.004	0.013	0.000	0.030					
NAN	0.000	0.000	0.000	0.000	0.000	0.000	0.000	0.000	0.000	0.000	0.000	0.000	0.000	0.000	**0.077**				
RAP	0.000	0.000	0.000	0.000	0.000	0.000	0.000	0.000	0.000	0.000	0.000	0.000	0.000	0.000	0.006	0.000			
CHO	0.000	0.000	0.000	0.000	0.000	0.000	0.000	0.000	0.000	0.000	0.000	0.000	0.000	0.000	0.000	0.000	0.000		
ROA	0.000	0.000	0.000	0.000	0.000	0.000	0.000	0.000	0.000	0.000	0.000	0.000	0.000	0.000	0.000	0.000	0.000	**0.456**	
ALL	0.000	0.000	0.000	0.000	0.000	0.000	0.000	0.000	0.000	0.000	0.000	0.000	0.000	0.000	0.000	0.000	0.000	**0.292**	**0.135**

Instances of nonsignificant (*P* > 0.05) genic heterogeneity are in bold.

**Table 3:** Probability values for pairwise tests of genic heterogeneity among blueback herring populations

	EMA	STG	EXE	MYS	MON	GIL	CON	HUD	DEL	NAN	JAM	RAP	CHO	ROA	NEU	CFE	SAN	ALT	SAV
STG	0.000																		
EXE	0.000	0.000																	
MYS	0.000	0.000	0.000																
MON	0.000	0.000	0.000	0.000															
GIL	0.000	0.000	0.000	0.000	0.000														
CON	0.000	0.000	0.000	0.000	0.000	0.000													
HUD	0.000	0.000	0.000	0.000	0.000	0.000	0.000												
DEL	0.000	0.000	0.000	0.000	0.000	0.000	0.000	0.000											
NAN	0.000	0.000	0.000	0.000	0.000	0.000	0.000	**0.126**	**0.671**										
JAM	0.000	0.000	0.000	0.000	0.000	0.000	0.000	0.000	0.000	**0.571**									
RAP	0.000	0.000	0.000	0.000	0.000	0.000	0.000	**0.072**	**0.044**	**0.794**	0.001								
CHO	0.000	0.000	0.000	0.000	0.000	0.000	0.000	0.000	0.000	**0.273**	0.000	0.003							
ROA	0.000	0.000	0.000	0.000	0.000	0.000	0.000	0.001	**0.091**	**0.418**	**0.117**	**0.060**	0.017						
NEU	0.000	0.000	0.000	0.000	0.000	0.000	0.000	0.000	0.000	0.025	0.001	0.013	0.010	**0.603**					
CFE	0.000	0.000	0.000	0.000	0.000	0.000	0.000	0.000	0.000	0.000	0.000	0.000	0.000	0.000	0.000				
SAN	0.000	0.000	0.000	0.000	0.000	0.000	0.000	0.000	0.000	0.000	0.000	0.000	0.000	0.000	0.000	0.000			
ALT	0.000	0.000	0.000	0.000	0.000	0.000	0.000	0.000	0.000	0.000	0.000	0.000	0.000	0.000	0.000	0.000	0.006		
SAV	0.000	0.000	0.000	0.000	0.000	0.000	0.000	0.000	0.000	0.000	0.000	0.000	0.000	0.000	0.000	0.000	0.000	0.008	
STJ	0.000	0.000	0.000	0.000	0.000	0.000	0.000	0.000	0.000	0.000	0.000	0.000	0.000	0.000	0.000	0.000	0.000	0.000	0.000

Instances of nonsignificant (*P* > 0.05) genic heterogeneity are in bold.

For alewife, standardized pairwise estimates of genetic differentiation (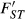) ranged from −0.003 to 0.352 (*F*_ST_ = −0.002 to 0.148) (Table S3); multilocus global 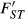
 = 0.119 (*F*_ST_ = 0.049). Nonsignificant (*P* > 0.05) genetic differentiation was observed primarily among pairwise comparisons of neighboring and geographically proximal alewife populations (Table S3). For blueback herring, 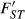 ranged from −0.008 to 0.233 (*F*_ST_ = −0.003 to 0.106) (Table S4); multilocus global 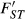
 = 0.067 (*F*_ST_ = 0.030). Nonsignificant (*P* > 0.05) genetic differentiation was observed predominately (27/28) among pairwise comparisons of neighboring and geographically proximal blueback herring populations in the center of the species’ range (Table S4).

For both species, hierarchical amova revealed a significant (*P* < 0.05) proportion of genetic variance partitioned among populations, and among individuals within populations (Table S5). Nonsignificant variation among temporal replicates for both alewife and blueback herring suggested stable population structure over at least short (i.e., 1–2 years) temporal scales.

#### Relationships among populations

**Figure 2:**
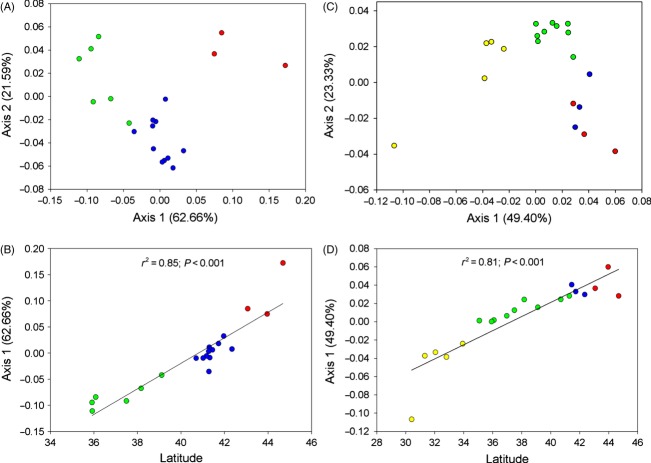
Results of principal coordinates analysis (PCoA) of multilocus microsatellite data for alewife (A, B) and blueback herring (C, D). Populations are color coded according to stock designations: Northern New England (red), Southern New England (blue), Mid-Atlantic (green), and South Atlantic (yellow). For both species, there is a significant relationship between latitude and PCoA Axis 1, indicating an effect of geography on patterns of population differentiation.

For alewife, PCoA revealed three factors that explained 92.25% of the variation in genetic distance (*D*_A_) among populations (Fig. 2A). Axis-1 explained 62.66% of this variation, and linear regression revealed a significant (*r*^2^ = 0.85; *P* < 0.001) relationship with latitude (Fig. 2B). For blueback herring, three factors explained 85.66% of the variation in genetic distance (*D*_A_) among populations (Fig. 2C). Axis-1 explained 49.40% of this variation, and linear regression revealed a significant (*r*^2^ = 0.81; *P* < 0.001) relationship with latitude (Fig. 2D).

#### Population structure

**Figure 3:**
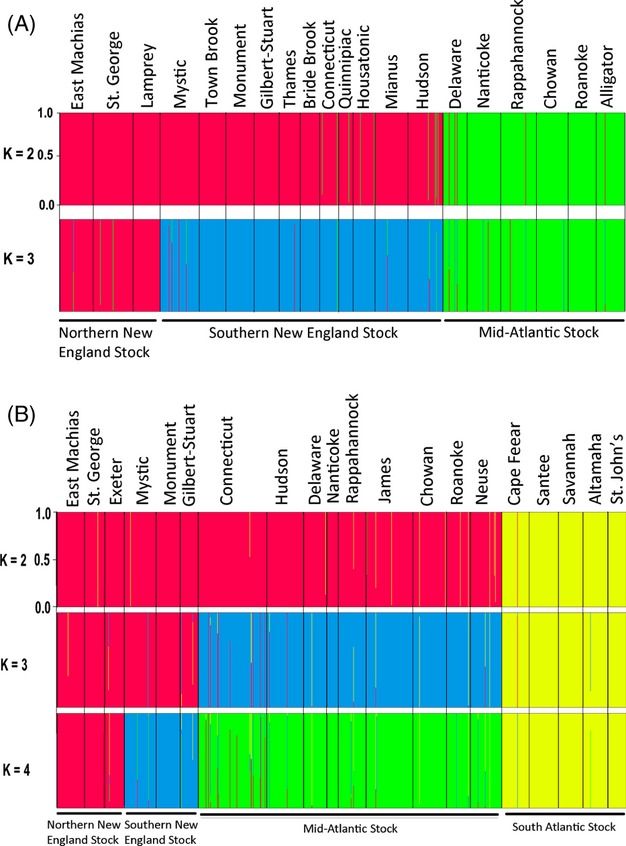
Alewife and blueback herring population structure and stock delineation inferred from Bayesian analyses. Individual specimens are indicated by a thin vertical line, which is partitioned into K-colored segments representing a specimen's estimated assignment fraction to each cluster. For alewife (A), analyses identified the most likely number of clusters at *K* = 3. For blueback herring (B), analyses identified the most likely number of clusters at *K* = 4.

For alewife, the maximum value of lnPr(X|*K*) using STRUCTURE was observed at *K *=* *4 (−24465.20). However, this estimate was only slightly greater than at *K* = 3 (−24470.13) but had considerably more variation, suggesting that *K* = 3 was more accurate (Fig. S1a). BAPS corroborated this result with significant (*P* < 0.001) support for three genetically distinguishable clusters. Both methods identified the same three clusters (hereafter referred to as stocks): Northern New England, Southern New England, and Mid-Atlantic (Fig. 3A). Further investigation using hierarchical STRUCTURE (Vaha et al. 2007) and BAPS analyses failed to detect additional structure within any of these stocks. Estimates of Δ*K* revealed the largest increase in the likelihood of the number of clusters at *K *=* *2 (Fig. S1a). amova revealed more variation among these three stocks (4.70%; *P* < 0.001) than among rivers within stock (1.30%; *P* < 0.001) (Table S5). The detection of significant variation among rivers within stocks is consistent with the significant genic differentiation detected among most populations (Table 2).

For blueback herring, the maximum value of lnPr(X|*K*) using STRUCTURE was observed at *K *= 6 (−35108.260). However, this estimate was only slightly greater than when *K* = 4 (−35189.77), or *K* = 5 (−35163.20) (Fig. S1b). BAPS had some difficulty resolving population structure and provided nearly equivalent support for either *K* = 4 (*P* = 0.503) or *K* = 5 (*P* = 0.497). However, the greater variation in estimates for *K* = 5 (Fig. S1b) suggests four clusters across the US range for blueback herring. Both STRUCTURE and BAPS identified the same four clusters (hereafter referred to as stocks): Northern New England, Southern New England, Mid-Atlantic, and South Atlantic (Fig. 3B). At *K* = 5, the St Johns separated from the South Atlantic Stock to represent a distinct cluster, as also suggested by PCoA (Fig. 2C, D). Further investigation using hierarchical STRUCTURE and BAPS analyses failed to detect additional structure within stocks. Estimates of Δ*K* revealed the largest increase in the likelihood of the number of clusters at *K* =* *2 (Fig. S1b) and suggested ‘deep-rooted’ structure among the populations surveyed. amova revealed more variation among the four stocks (2.45%; *P* < 0.001) than among rivers within stocks (0.82%; *P* < 0.001) and was comparable with the among river component of variation (3.21%, *P* < 0.05) when populations were not grouped into stocks (Table S5). That amova detected significant variation among rivers within stocks was consistent with the significant genic differentiation observed among most populations sampled (Table 3).

#### Isolation by distance

**Figure 4:**
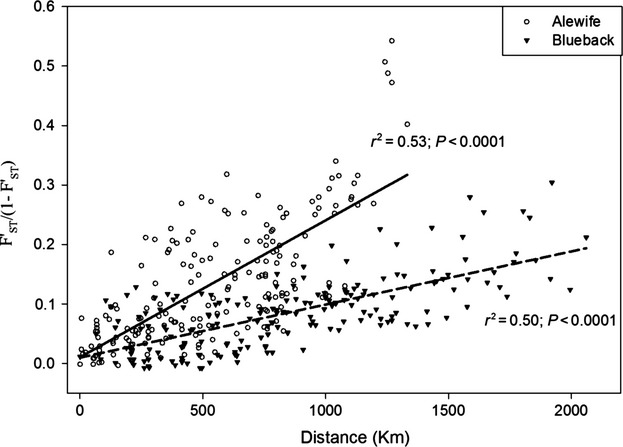
Isolation by distance (IBD) relationships for alewife and blueback herring. Both species show significant IBD, with alewife displaying a steeper slope of the relationship, indicating less gene flow among alewife populations.

Mantel tests revealed a highly significant (*P* < 0.001) pattern of IBD for both alewife (*r* = 0.73) and blueback herring (*r* = 0.71) across their US range. The slope of the IBD relationship was steeper in alewife (slope = 2.3 e-4) compared with blueback herring (slope = 8.9 e-5), suggesting greater genetic isolation among alewife populations or, conversely, more gene flow among blueback herring populations (Fig. 4).

### Demographic analysis

#### Demographic trends by time series

Time series revealed an overall pattern of demographic declines in alewife and blueback herring. For alewife, of a total of 40 time series analyzed, 11 showed significant declines, 16 showed nonsignificant declines, 2 showed no change, 10 showed nonsignificant increases and 1 showed a significant increase (Table S6). Mann–Kendall tests revealed that mean length for spawning adult alewives has declined significantly in 4 of 10 rivers examined (Stony Brook, Monument, Hudson, and Chowan; Fig. S2), and results were similar for males and females (Table S6). Alewife run size declined significantly in 3 of 20 rivers examined (Parker, Nonquit, and Chowan; Fig. S3) and increased significantly in one river (York; Fig. S3, Table S6).

Of a total of 29 time series analyzed for blueback herring, 18 showed significant declines, six showed nonsignificant declines, one showed no change, three showed nonsignificant increases, and none showed significant increases (Table S7). Mann–Kendall tests revealed that mean length for spawning adult blueback herring has declined significantly in seven of nine rivers examined (Oyster, Monument, Hudson, Nanticoke, Chowan, Santee and St. Johns; Fig. S4). Results were similar for males and females with the exception of the St. Johns, for which declines were significant for females only (Table S7). Blueback herring run size declined significantly in four of nine rivers examined (Monument, Shetucket, Chowan, and Cooper; Fig. S5, Table S7).

#### Demographic trends by species and stock

**Figure 5:**
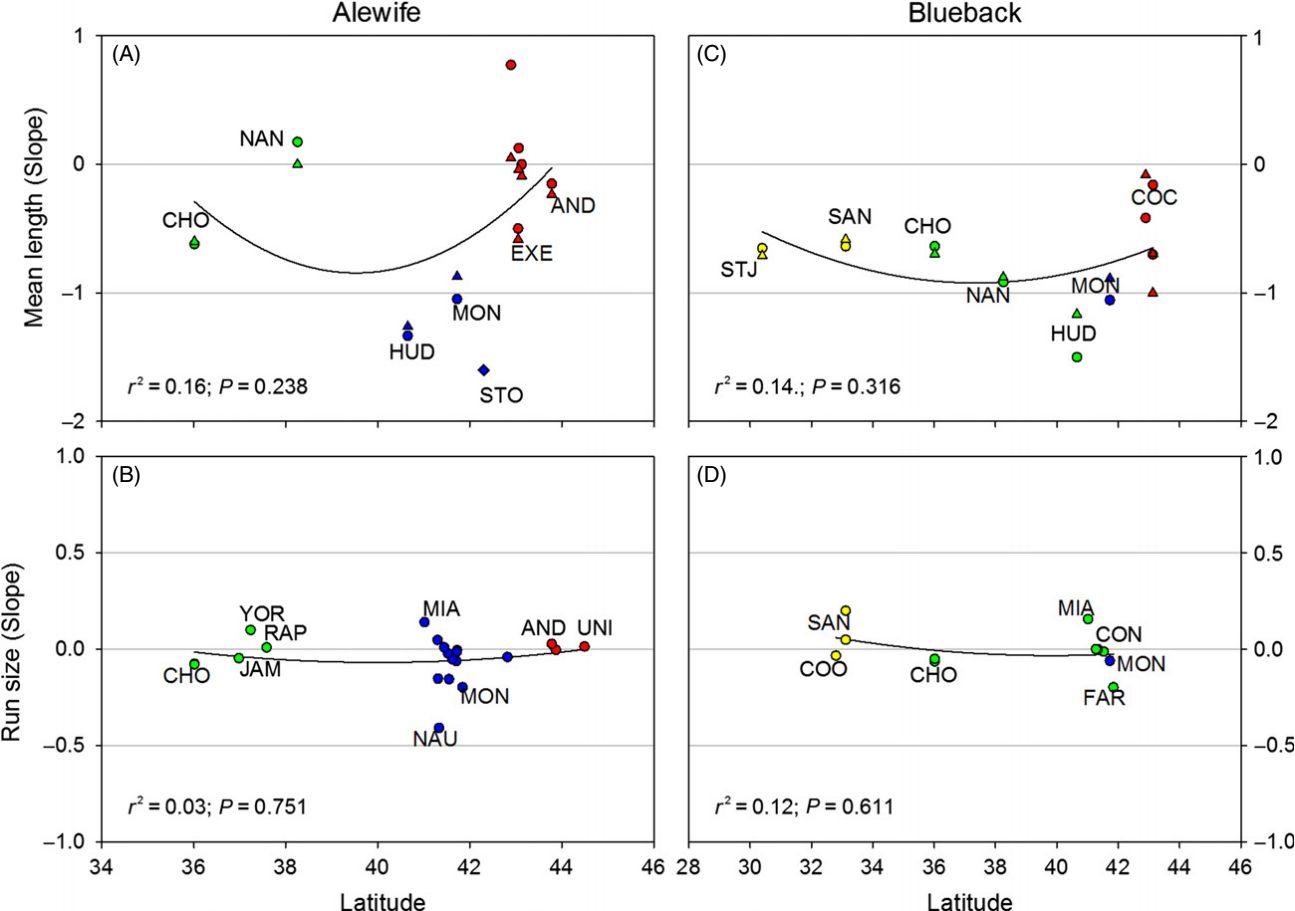
Slope values estimated from demographic time series for alewife (A, B) and blueback herring (C, D) plotted against latitude and color coded by stock: Northern New England (red), Southern New England (blue), Mid-Atlantic (green), South Atlantic (yellow). River codes are given for a subset of the time series analyzed. Negative slopes indicate declines over time. For mean length of spawning adults, slopes were estimated separately for males (triangles) and females (circles), with one exception where the sexes were grouped (diamond). Quadratic linear regressions show the tendency for declines to be more severe at the center of the sampled distribution, especially for mean length.

Time series clearly show declines over time and general linear models revealed significant differences in the magnitude of declines between species and among stocks. For both species, all stocks showed average declines in mean length and run size over time (i.e., although a few individual rivers increased, the average trend for all stocks was negative). Overall, declines have been most dramatic in the central portions of each species range, especially for mean length of spawning adults (Fig. 5).

When comparing between species, the mean length of spawning adults has declined significantly more in blueback herring compared with alewife (*F*_1, 35_ = 4.159, *P* = 0.049; Fig. 5A, C). Declines in adult run counts over time did not differ between the species (*F*_1, 30_ = 1.158, *P* = 0.290; Fig. 5B, D).

For alewife, changes in mean length differed significantly among stocks (*F*_2, 14_ = 12.558, *P* = 0.001), with the Southern New England Stock showing more dramatic declines than either the Northern New England Stock (Tukey's HSD: *P* = 0.001) or the Mid-Atlantic Stock (Tukey's HSD: *P* = 0.011) (Fig. 5A; Fig. S2). Changes in the mean length of spawning adult alewives did not differ between females and males (*F*_1, 14_ = 0.474, *P* = 0.503). Declines in mean alewife run size were evident across all stocks but did not differ among stocks (*F*_2, 18_ = 0.799, *P* = 0.465) (Fig. 5B; Fig. S3).

For blueback herring, changes in mean length showed marginally significant differences among stocks (*F*_3, 13_ = 2.861, *P* = 0.078), with the Southern New England and Mid-Atlantic Stocks declining more steeply than the Northern New England and Southern Atlantic Stocks (although Tukey's HSD did not reveal any pairwise differences to be significant) (Fig. 5C; Fig. S4). Declines in the mean length of spawning adult blueback herring did not differ between females and males (*F*_1, 13_ = 0.001, *P* = 0.981). Declines in blueback herring run size were observed across all stocks but did not differ among stocks (*F*_2, 8_ = 0.978, *P* = 0.417) (Fig. 5D; Fig. S5).

#### Conservation prioritization

For alewife stock-level prioritizations, the Southern New England Stock was designated as high priority and the Northern New England and Mid-Atlantic Stock were designated as medium priority. Conservation prioritization of specific rivers within stocks highlights the genetic distinctiveness observed among populations. At the population level (for a total of 45 alewife populations), six populations were designated as low priority, 23 as medium priority, and 15 as high priority (Table 4). High-priority populations are located in the middle of the US range, with the addition of several high-priority populations at the extreme southern end of the alewife distribution. At this end of the distribution, the Roanoke and Alligator were given high prioritizations due to genetic similarity to the Chowan, which has declined dramatically (Fig. 5; Table S6). For blueback herring stock-level prioritizations, the Southern New England and Mid-Atlantic Stocks were designated as high priority, and the Northern New England and South Atlantic Stocks were designated as medium priority. At the population level (for a total of 55 blueback herring populations), 0 populations were designated as low priority, 26 as medium priority, and 29 as high priority (Table 4). High-priority blueback herring stocks and populations are located in the middle of the US range, with the addition of the St Jonhs in Florida. This population was given high prioritization due to its genetic uniqueness (Fig. 2) and declines observed for mean length (Fig. 5; Table S7).

**Table 4:** Conservation prioritizations for alewife and blueback herring populations

		Alewife			Blueback herring		
State	River	Demographic data	Genetic stock	Prioritization	Demographic data	Genetic stock	Prioritization
ME	Dennys	N	NNE	Medium	N	NNE	Medium
ME	East Machias	N	NNE	Medium	N	NNE	Medium
ME	Narraguagus	N	NNE	Medium	N	NNE	Medium
ME	Union	Y	NNE	Low	N	NNE	Medium
ME	Orland	N	NNE	Medium	N	NNE	Medium
ME	Penobscot	N	NNE	Medium	N	NNE	Medium
ME	Soudabscook	N	NNE	Medium	N	NNE	Medium
ME	St George	N	NNE	Medium	N	NNE	Medium
ME	Damariscotta	Y	NNE	Medium	N	NNE	Medium
ME	Sheepscot	N	NNE	Medium	N	NNE	Medium
ME	Kennebec	N	NNE	Medium	N	NNE	Medium
ME	Androscoggin	Y	NNE	Medium	N	NNE	Medium
ME	Presumpscot	N	NNE	Medium	N	NNE	Medium
ME	Saco	N	NNE	Medium	N	NNE	Medium
NH	Cocheco	Y	NNE	Medium	Y	NNE	Medium
NH	Oyster	N	NNE	Medium	Y	NNE	High
NH	Exeter	Y	NNE	Medium	N	NNE	Medium
NH	Lamprey	Y	NNE	Low	N	NNE	Medium
NH	Winnicut	Y	NNE	Low	Y	NNE	Medium
MA	Merrimac	N	SNE	High	N	SNE	High
MA	Parker	Y	SNE	Medium	N	SNE	High
MA	Mystic	N	SNE	High	N	SNE	High
MA	Charles	N	SNE	High	N	SNE	High
MA	Stony Brook	Y	SNE	High	N	SNE	High
MA	Town Brook	N	SNE	High	N	SNE	High
MA	Monument	Y	SNE	High	Y	SNE	High
MA	Mattipoisett	Y	SNE	High	N	SNE	High
MA	Nemasket	Y	SNE	High	N	SNE	High
RI	Nonquit	Y	SNE	High	N	SNE	High
RI	Gilbert Stuart	Y	SNE	Low	N	SNE	High
CT	Connecticut	N	SNE	High	Y	MAT	Medium
CT	Quinnipiac	N	SNE	High	N	MAT	High
CT	Housatonic	N	SNE	High	N	MAT	High
NY	Hudson	Y	SNE	High	Y	MAT	High
NJ	Raritan	N	MAT	Medium	N	MAT	High
NJ/DE/PA	Delaware	N	MAT	Medium	N	MAT	High
MD	Nanticoke	Y	MAT	Medium	Y	MAT	High
MD	Susquehanna	N	MAT	Medium	N	MAT	High
MD/VA	Potomac	N	MAT	Medium	N	MAT	High
VA	Rappahannock	Y	MAT	Low	N	MAT	High
VA	York	Y	MAT	Low	N	MAT	High
VA	James	Y	MAT	Medium	N	MAT	High
NC	Chowan	Y	MAT	High	Y	MAT	High
NC	Roanoke	N	MAT	High	N	MAT	High
NC	Alligator	N	MAT	High	N	MAT	High
NC	Tar-Pamlico	–	–	–	N	MAT	High
NC	Neuse	–	–	–	N	MAT	High
NC	Cape Fear	–	–	–	N	SAT	Medium
SC	Pee Dee	–	–	–	N	SAT	Medium
SC	Santee	–	–	–	Y	SAT	Medium
SC	Cooper	–	–	–	Y	SAT	Medium
SC	Edisto	–	–	–	N	SAT	Medium
SC/GA	Savannah	–	–	–	N	SAT	Medium
GA	Altamaha	–	–	–	N	SAT	Medium
FL	St Johns	–	–	–	Y	SAT	High

For each population, the availability of demographic data and genetic stock assignments are given: Stocks = Northern New England (NNE), Southern New England (SNE), Mid-Atlantic (MAT), and South Atlantic (SAT).

## Discussion

We analyzed population genetic structure and recent demographic trends in anadromous alewife and blueback herring to designate management units and prioritize populations within those units for conservation efforts. Our results show that the majority of rivers examined comprise genetically distinguishable groups (Tables 2 and 3). This finding is consistent with microsatellite studies of other anadromous alosine species (Jolly et al. 2012; Hasselman et al. 2013). For alewife, notable exceptions to this pattern (i.e., rivers showing nonsignificant genic differentiation) include some rivers associated with Long Island Sound (see also Palkovacs et al. 2008) and Albemarle Sound (Table 2). For blueback herring, instances of nonsignificant genic differentiation are found in the middle of the range, with most occurring in the vicinity of Chesapeake Bay (Table 3). The higher frequency of nonsignificantly differentiated rivers found for blueback herring is supported by isolation-by-distance (IBD) patterns, which also suggest greater gene flow among blueback herring populations (Fig. 4). The finding of significant differentiation among most rivers suggests that alewife and blueback herring should be managed at the river-level where possible, with the possible exceptions of Long Island Sound and Albemarle Sound for alewife, and Chesapeake Bay for blueback herring, which could be managed as units.

Our results indicate the presence of three distinct genetic stocks in alewife and four distinct genetic stocks in blueback herring (Figs 2 and 3). The presence of high-level population genetic structure indicates that gene flow is not continuous across all parts of these species ranges. In alewife, genetic stocks include a Northern New England Stock, a Southern New England Stock, and a Mid-Atlantic Stock (Fig. 3A). In blueback herring, genetic stocks include a Northern New England Stock, a Southern New England Stock, a Mid-Atlantic Stock, and a South Atlantic Stock (Fig. 3B). There is a high level of congruence between what *F*_ST_-based methods (Tables 2, 3, S3 and S4) and Bayesian clustering methods (Fig. 3) identify as genetically distinguishable stocks. Thus, we have confidence that we have identified the major genetic stocks within the US portions of these species ranges.

Demographic information for alewife and blueback herring exists for a relatively small number of populations. We analyzed existing data for mean length of spawning adults and spawning adult run size in the context of genetic stock structure. This analysis reveals that declines have occurred across all stocks. Overall, variation between populations and stocks was greater for mean length data compared with run size data (Fig. 5). The magnitude of declines has been greater in blueback herring compared with alewife, especially for mean length, and most severe toward the center of each species US range (between about 40–42°N latitude for both species; Fig. 5).

In alewife, declines have been most dramatic and widespread for the Southern New England Stock. We recommend high conservation prioritization for most alewife populations in this stock (Table 4). Although the Mid-Atlantic Stock has performed somewhat better, alewife populations associated with Albemarle Sound (Chowan, Roanoke, Alligator) were given high conservation priority due to dramatic declines observed in the genetically similar Chowan (Figs 4, S3 and S4). A possible southern range contraction in alewife puts these Albemarle Sound populations at particular risk. Compared with other alewife stocks, the Northern New England alewife stock is performing relatively well, with some populations remaining stable and some even showing recent (albeit modest) hints of recovery (Figs 4, S3 and S4).

In blueback herring, declines have been most severe and widespread for the Southern New England and Mid-Atlantic Stocks. We recommend high conservation prioritization for most blueback herring populations belonging to these stocks (Table 4). The Northern New England and South Atlantic Stocks appear to have declined less dramatically. Nonetheless, the St Johns in Florida was given high prioritization due to its genetic uniqueness, declines observed in mean length, and vulnerable location at the extreme southern end of the blueback herring range. It is important to note that demographic information for blueback herring populations is particularly limited. For example, demographic information for the Northern New England and South Atlantic Stocks is limited to just three rivers per stock, and demographic information for the Southern New England Stock is limited to just a single river. Expansion of data collection efforts for river herring, particularly for blueback herring, is critical for setting and achieving future conservation goals.

Recent alewife and blueback herring declines may have been triggered by overharvest in marine fisheries, but earlier human actions including in-river harvest, dam construction, pollution, and landscape change undoubtedly reduced the resiliency of populations (Limburg and Waldman 2009; Hall et al. 2012). Current threats include marine bycatch, rebounding populations of natural predators, urbanization of coastal watersheds, climate change, and changes to marine ecosystems (ASMFC 2012). Recent restoration efforts such as fishway projects on main stem dams of large rivers have largely failed to increase populations (Brown et al. 2013). We recommend systematic monitoring and evaluation of ongoing freshwater restoration projects and increased focus on marine processes. A major emerging concern is bycatch in marine fisheries, which overlaps geographically with regions we found to be declining most precipitously (Bethoney et al. 2013; Cournane et al. 2013).

Our findings have important implications for managing interbasin transfers of gravid adults, a strategy that is being increasingly implemented in the name of alewife and blueback herring restoration (Hasselman and Limburg 2012). Interbasin transfers should not occur across major stock or watershed boundaries for either species. Higher straying rates inferred for blueback herring (Fig. 4) make the effects of stocking across drainages perhaps less disruptive for population structure in this species. However, greater straying also makes natural recolonization of watersheds more likely (and hence stocking less necessary to re-establish spawning runs). Interbasin transfers will be least disruptive to population structure in river complexes not showing significant differentiation, including Long Island Sound and Albemarle Sound for alewife and Chesapeake Bay for blueback herring. However, interbasin transfers may still disrupt local adaptation even when neutral genetic structure is minimal, an effect which may be hindering the recovery of American shad (*Alosa sapidissima*) (Hasselman and Limburg 2012). Thus, interbasin stocking should be used judiciously, for the re-establishment of extirpated runs, and source populations should be as geographically proximate as possible.

We combined genetic and demographic information to define management units and prioritize populations within those units for conservation action. The rationale for this approach is based on the fact that population genetic structure is the legacy of demographic nonindependence caused by migration. Specifically, linking ‘evolutionary measures’ of population genetic structure and ‘ecological measures’ of demographic nonindependence remain challenging because the power to detect population structure using genetic data varies between methods and marker types (Waples and Gaggiotti 2006). Nonetheless, our results show that this approach can be useful, especially when demographic information must be generalized from just a few populations and conservation decisions are urgent, as is the case for anadromous alewife and blueback herring.

## Data archiving

Microsatellite data are available from the DRYAD Digital Repository: http://dx.doi.org/10.5061/dryad.8v0c3.

## Supporting Information

**Figure S1**. Bayesian inference of the number of clusters (K) among populations sampled for alewife (a) and blueback herring (b) using plateau of log probability of data L(K) (• ± SD; Pritchard et al. 2000) and DK (⋆; Evanno et al. 2005). **Figure S2**. Alewife time series data for mean length of spawning adult females for the Northern New England Stock (a), Southern New England Stock (b), and Mid-Atlantic Stock (c). **Figure S3**. Alewife time series data for run size for the Northern New England Stock (a), Southern New England Stock (b), and Mid-Atlantic Stock (c). **Figure S4**. Blueback herring time series data for mean length of spawning adult females for the Northern New England Stock (a), Southern New England Stock (b), and Mid-Atlantic Stock (c), and South Atlantic Stock. **Figure S5**. Blueback herring time series data for run size for the Southern New England Stock (a), and Mid-Atlantic Stock (b), and South Atlantic Stock (c).

**Table S1**. Alewife genetic diversity statistics: number of specimens genotyped (N), number of alleles per locus (N_a_), allelic richness (R; standardized to N = 24), observed heterozygosity (H_O_), expected heterozygosity (H_E_), and inbreeding coefficient (F_IS_). **Table S2**. Blueback herring genetic diversity statistics: number of specimens genotyped (N), number of alleles per locus (Na), allelic richness (R standardized to N = 26), observed heterozygosity (HO), expected heterozygosity (HE), and inbreeding coefficient (FIS). **Table S3**. Alewife genetic differentiation. Pairwise F_ST_ values (h; Weir and Cockerham 1984) below diagonal (nonsignificant values in bold) and standardized F_ST_ values (F0ST; Hedrick 2005) above diagonal. **Table S4**. Blueback herring genetic differentiation. Pairwise F_ST_ values (h; Weir and Cockerham 1984) below diagonal (non-significant values in bold) and standardized F_ST_ values (F_ST_; Hedrick 2005) above diagonal. **Table S5**. AMOVA results. Clusters refer to genetic stocks identified using STRUCTURE v.2.3.3 (Pritchard et al. 2000; Falush et al. 2003) and BAPS v.5.3 (Corander et al. 2006). **Table S6**. Alewife demographic time series results with genetic stock assignments listed for each river (NNE-Northern New England, SNESouthern New England, MAT-Mid-Atlantic). Non-parametric linear regression slopes are given (significant values in bold). **Table S7**. Blueback herring demographic time series results with genetic stock assignments listed for each river (NNE-Northern New England, SNE-Southern New England, MAT-Mid-Atlantic, SAT-South Atlantic). **Table S8**. Organizations and individuals that provided assistance with sample collection.
