# Supplementary material for: Combining genetic and demographic information to prioritize conservation efforts for anadromous alewife and blueback herring
Source: Evol Appl. 2013 Oct 2;7(2):212–26. doi: 10.1111/eva.12111 (PMC3927884; doi:10.1111/eva.12111)
Supplement: Supplementary file 1 — Figure S1. Bayesian inference of the number of clusters (K) among populations sampled for alewife (a) and blueback herring (b) using plateau of log probability of data L(K) (• ± SD; Pritchard et al. 2000) and DK (⋆; Evanno et al. 2005). Figure S2. Alewife time series data for mean length of spawning adult females for the Northern New England Stock (a), Southern New England Stock (b), and Mid-Atlantic Stock (c). Figure S3. Alewife time series data for run size for the Northern New England Stock (a), Southern New England Stock (b), and Mid-Atlantic Stock (c). Figure S4. Blueback herring time series data for mean length of spawning adult females for the Northern New England Stock (a), Southern New England Stock (b), and Mid-Atlantic Stock (c), and South Atlantic Stock. Figure S5. Blueback herring time series data for run size for the Southern New England Stock (a), and Mid-Atlantic Stock (b), and South Atlantic Stock (c). [file eva0007-0212-sd1.pdf]

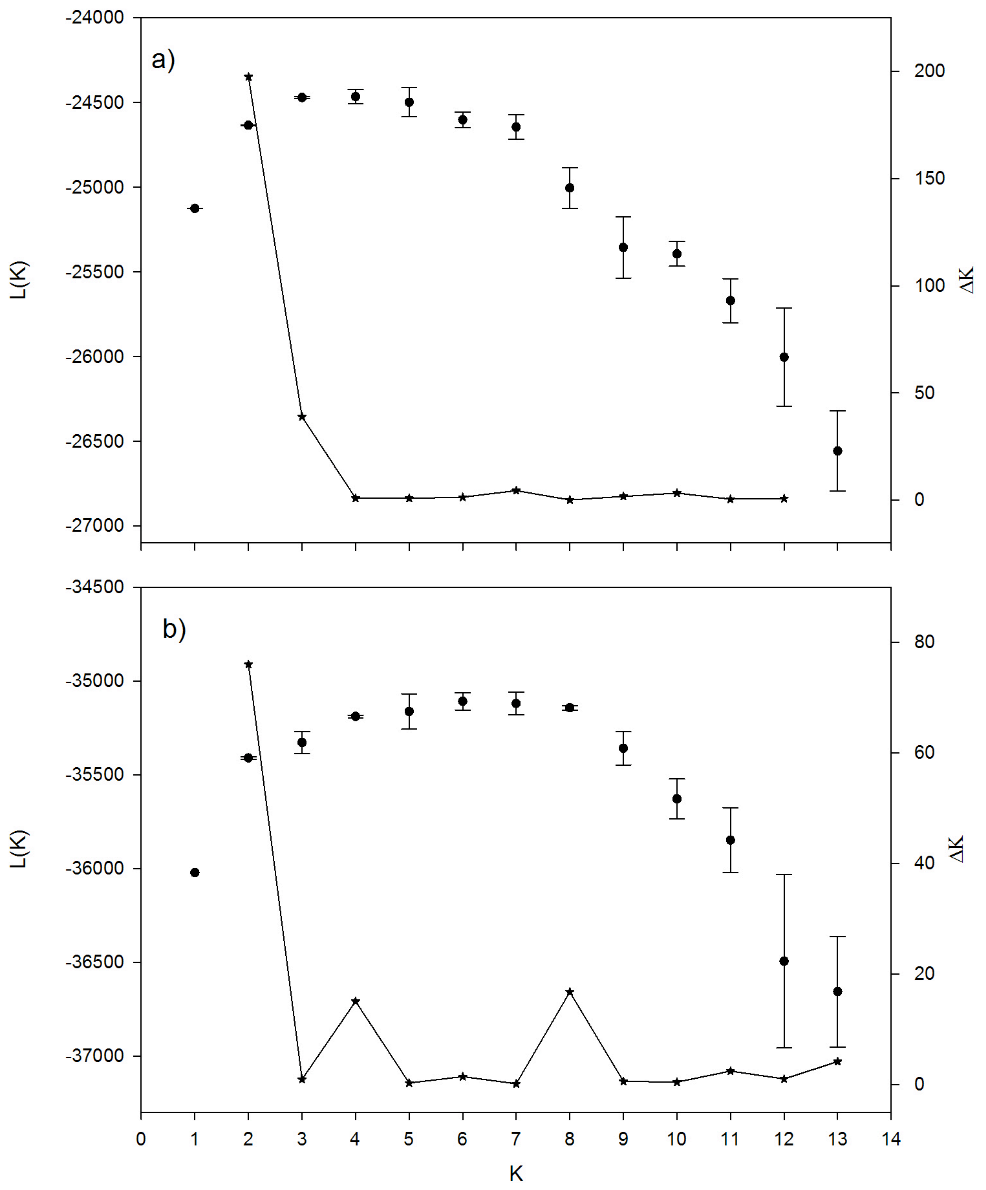

Figure S1: Bayesian inference of the number of clusters (K) among populations sampled for alewife (a) and blueback herring (b) using plateau of log probability of data L(K) (• ± sd; Pritchard et al. 2000) and ΔK (★; Evanno et al. 2005). Applying the criteria from these methods suggested K=3 clusters for alewife and K=4 clusters for blueback herring.

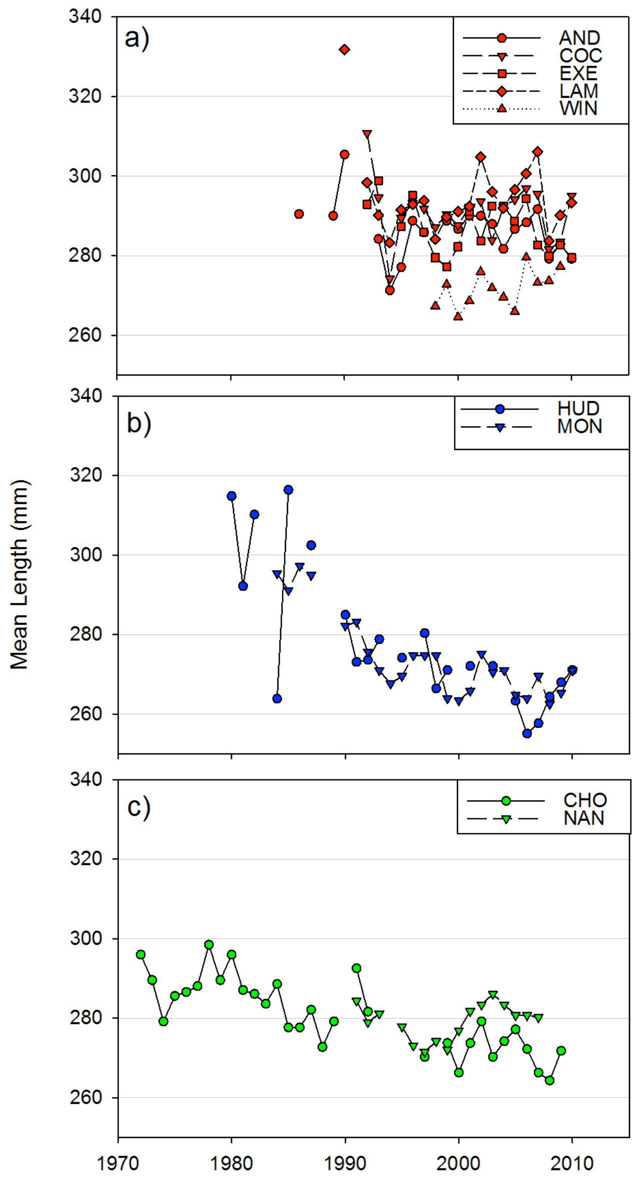

Figure S2: Alewife time series data for mean length of spawning adult females for the Northern New England Stock (a), Southern New England Stock (b), and Mid-Atlantic Stock (c). Declines are statistically significant for the Monument (MON), Hudson (HUD) and Chowan (CHO).

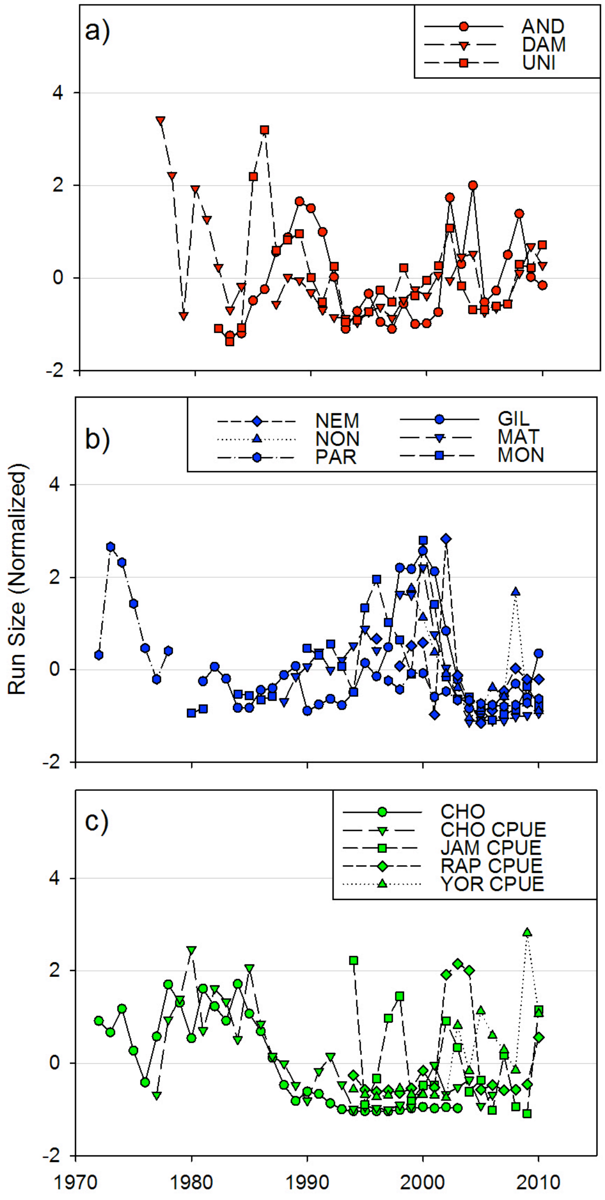

Figure S3: Alewife time series data for run size for the Northern New England Stock (a), Southern New England Stock (b), and Mid-Atlantic Stock (c). Only time series with >10 years of data are shown, which excluded 6 rivers in the Southern New England Stock. Declines are statistically significant for the Parker (PAR), Nonquit (NON), and Chowan (CHO). A statistically significant increase in run size is indicated in the York (YOR).

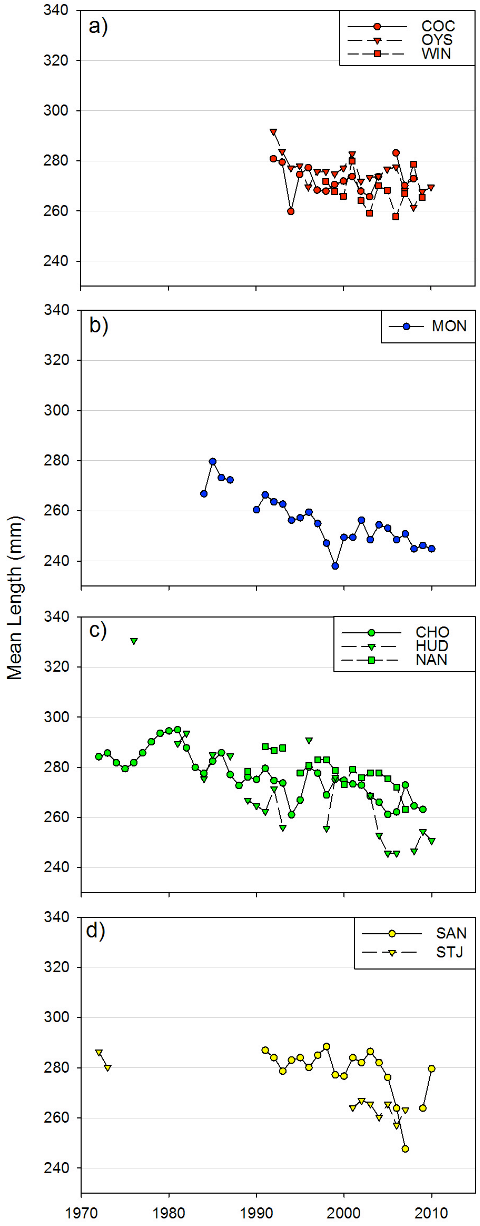

Figure S4: Blueback herring time series data for mean length of spawning adult females for the Northern New England Stock (a), Southern New England Stock (b), and Mid-Atlantic Stock (c), and South Atlantic Stock. Declines are statistically significant for the Oyster (OYS), Monument (MON), Hudson (HUD), Nanticoke (NAN), Chowan (CHO), Santee (SAN), and St. Johns (STJ).

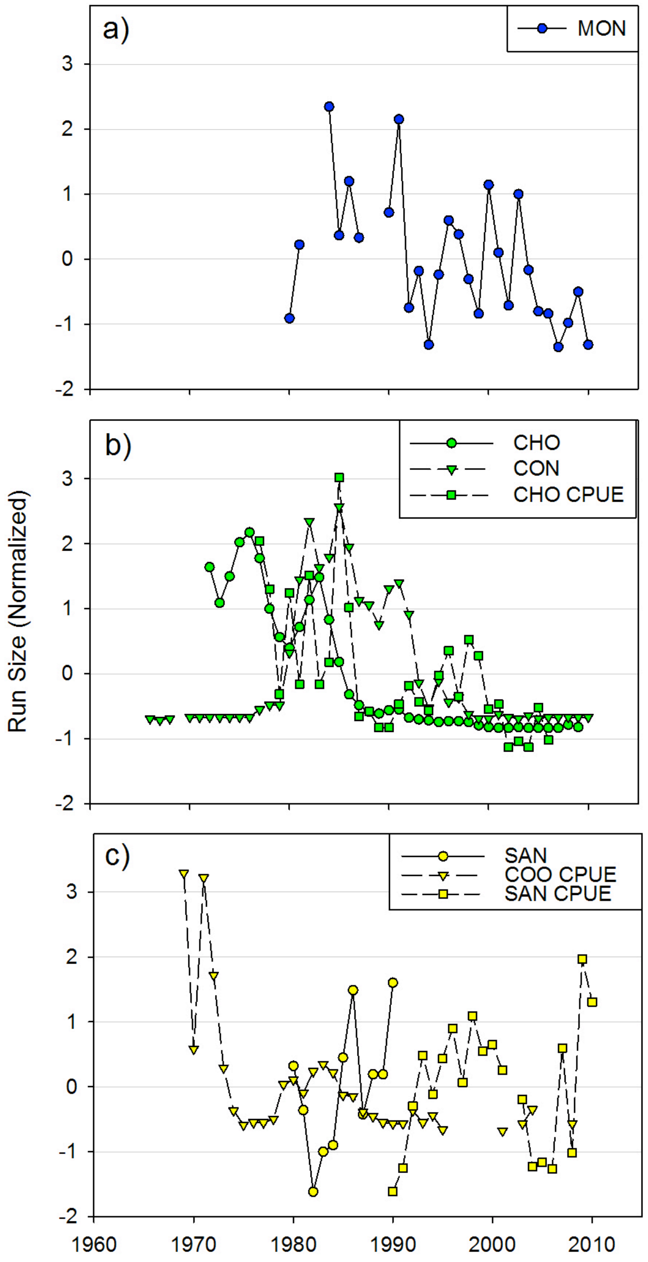

Figure S5: Blueback herring time series data for run size for the Southern New England Stock (a), and Mid-Atlantic Stock (b), and South Atlantic Stock (c). No run size data were available for the blueback Northern New England Stock. Declines are statistically significant for the Monument (MON), Shetucket (SHE), Chowan (CHO), and Cooper (COO).
