# Supplementary material for: Combining genetic and demographic information to prioritize conservation efforts for anadromous alewife and blueback herring
Source: Evol Appl. 2013 Oct 2;7(2):212–26. doi: 10.1111/eva.12111 (PMC3927884; doi:10.1111/eva.12111)
Supplement: Supplementary file 2 — Table S1. Alewife genetic diversity statistics: number of specimens genotyped (N), number of alleles per locus (Na), allelic richness (R; standardized to N = 24), observed heterozygosity (HO), expected heterozygosity (HE), and inbreeding coefficient (FIS). Table S2. Blueback herring genetic diversity statistics: number of specimens genotyped (N), number of alleles per locus (Na), allelic richness (R standardized to N = 26), observed heterozygosity (HO), expected heterozygosity (HE), and inbreeding coefficient (FIS). Table S3. Alewife genetic differentiation. Pairwise FST values (h; Weir and Cockerham 1984) below diagonal (nonsignificant values in bold) and standardized FST values (F0ST; Hedrick 2005) above diagonal. Table S4. Blueback herring genetic differentiation. Pairwise FST values (h; Weir and Cockerham 1984) below diagonal (non-significant values in bold) and standardized FST values (FST; Hedrick 2005) above diagonal. Table S5. AMOVA results. Clusters refer to genetic stocks identified using STRUCTURE v.2.3.3 (Pritchard et al. 2000; Falush et al. 2003) and BAPS v.5.3 (Corander et al. 2006). Table S6. Alewife demographic time series results with genetic stock assignments listed for each river (NNE-Northern New England, SNESouthern New England, MAT-Mid-Atlantic). Non-parametric linear regression slopes are given (significant values in bold). Table S7. Blueback herring demographic time series results with genetic stock assignments listed for each river (NNE-Northern New England, SNE-Southern New England, MAT-Mid-Atlantic, SAT-South Atlantic). Table S8. Organizations and individuals that provided assistance with sample collection. [file eva0007-0212-sd2.pdf]

Table S1: Alewife genetic diversity statistics: number of specimens genotyped (N), number of alleles per locus (N_a), allelic richness (R; standardized to N=24), observed heterozygosity (H_O), expected heterozygosity (H_E), and inbreeding coefficient (F_{IS}).

			Population																			
**Locus**	**A**		**EMA**	**STG**	**LAM**	**MYS**	**MON**	**TOW**	**GIL**	**THA**	**BRI**	**CON**	**QUI**	**HOU**	**MIA**	**HUD**	**DEL**	**NAN**	**RAP**	**CHO**	**ROA**	**ALL**
Aa046	5	N	54	68	46	68	45	49	44	35	34	33	25	38	56	61	41	57	61	55	47	49
Range	200-228	N_a	2	2	2	2	2	2	2	3	2	3	2	3	3	2	2	2	2	2	2	2
		R	2.00	2.00	2.00	2.00	2.00	2.00	2.00	2.69	2.00	2.73	2.00	2.63	2.43	2.00	2.00	2.00	2.00	2.00	2.00	2.00
		F_{IS}	-0.01	-0.23	0.01	0.00	-0.14	0.30	-0.19	0.06	-0.06	0.26	-0.17	0.07	-0.26	-0.11	0.13	0.08	0.18	0.01	0.23	-0.13
		H_E	0.50	0.50	0.51	0.49	0.23	0.44	0.29	0.36	0.44	0.45	0.41	0.48	0.44	0.47	0.48	0.46	0.46	0.48	0.49	0.50
		H_O	0.50	0.62	0.50	0.49	0.27	0.31	0.34	0.34	0.47	0.33	0.48	0.45	0.55	0.52	0.41	0.42	0.38	0.47	0.38	0.57
Aa074	11	N	50	68	40	57	44	49	33	34	34	32	25	38	49	60	41	50	61	52	48	44
Range	206-250	N_a	8	8	7	8	7	8	6	7	6	7	7	7	5	8	7	9	10	7	8	8
		R	6.80	6.68	6.56	6.65	6.88	7.43	5.45	6.66	5.68	6.63	6.96	6.45	4.49	7.03	6.45	7.23	7.67	6.15	7.09	6.76
		F_{IS}	-0.09	-0.08	0.03	-0.05	-0.03	0.06	-0.02	0.14	-0.17	0.02	-0.02	0.03	-0.08	-0.05	-0.12	0.06	0.10	-0.03	0.11	0.00
		H_E	0.75	0.68	0.72	0.65	0.77	0.78	0.68	0.75	0.71	0.77	0.79	0.73	0.57	0.78	0.76	0.79	0.80	0.73	0.73	0.66
		H_O	0.82	0.74	0.70	0.68	0.80	0.73	0.70	0.65	0.82	0.75	0.80	0.71	0.61	0.82	0.85	0.74	0.72	0.75	0.65	0.66
Ap058	9	N	54	68	45	68	45	49	44	35	34	33	25	38	54	60	40	55	61	55	49	49
Range	249-265	N_a	2	3	2	4	3	5	5	5	5	5	5	5	5	5	4	5	7	4	4	5
		R	2.00	2.35	2.00	3.78	2.53	3.72	3.89	4.06	4.89	4.89	4.88	4.89	4.39	3.59	3.20	3.81	4.60	2.87	3.23	3.47
		F_{IS}	-0.10	0.06	-0.16	-0.03	-0.10	-0.04	0.09	-0.05	0.10	0.21	0.00	0.40	0.10	0.05	-0.20	-0.05	0.04	0.02	-0.29	-0.07
		H_E	0.45	0.50	0.50	0.53	0.46	0.41	0.55	0.52	0.62	0.61	0.56	0.61	0.60	0.53	0.52	0.54	0.50	0.43	0.40	0.50
		H_O	0.50	0.47	0.58	0.54	0.51	0.43	0.50	0.54	0.56	0.48	0.56	0.37	0.54	0.50	0.63	0.56	0.48	0.42	0.51	0.53
Ap071	11	N	56	65	39	65	39	49	40	35	34	32	24	38	56	58	41	51	60	49	41	44
Range	195-295	N_a	5	4	3	4	4	4	5	5	4	4	5	5	4	7	5	5	5	5	5	6
		R	3.86	3.81	3.00	3.90	4.00	4.00	4.82	4.66	3.92	4.00	5.00	4.63	4.00	5.48	4.59	4.40	4.40	4.48	4.59	5.34
		F_{IS}	0.06	0.09	-0.02	-0.08	0.10	0.18	0.08	0.31	-0.08	-0.03	0.08	-0.01	0.06	0.07	-0.03	0.23	0.06	-0.01	0.17	0.18
		H_E	0.51	0.49	0.48	0.63	0.71	0.69	0.71	0.70	0.68	0.70	0.77	0.65	0.70	0.69	0.71	0.54	0.64	0.69	0.70	0.75
		H_O	0.48	0.45	0.49	0.68	0.64	0.57	0.65	0.49	0.74	0.72	0.71	0.66	0.66	0.64	0.73	0.41	0.60	0.69	0.59	0.61
Ap038	7	N	57	65	39	44	42	47	42	36	33	32	25	38	57	59	42	49	41	46	49	46
Range	222-254	N_a	4	3	3	4	3	2	3	2	3	2	2	5	2	3	4	6	3	4	5	3
		R	3.28	2.37	2.62	3.09	2.57	2.00	2.82	2.00	2.73	2.00	2.00	3.90	2.00	2.41	3.57	5.35	3.00	3.77	4.23	2.90
		F_{IS}	-0.07	-0.05	0.02	0.09	0.24	0.08	0.08	-0.05	0.08	-0.15	0.52	0.18	-0.17	0.17	0.05	0.06	-0.03	0.00	-0.02	0.09
		H_E	0.18	0.26	0.39	0.42	0.16	0.21	0.41	0.32	0.36	0.49	0.25	0.35	0.42	0.37	0.53	0.63	0.60	0.57	0.64	0.52
		H_O	0.19	0.28	0.38	0.39	0.12	0.19	0.38	0.33	0.33	0.56	0.12	0.29	0.49	0.31	0.50	0.59	0.61	0.57	0.65	0.48
Ap010	19	N	58	65	46	61	42	49	44	34	32	33	25	38	56	60	40	53	57	49	47	48
Range	221-263	N_a	9	7	8	9	10	10	9	9	9	8	9	11	8	11	11	11	13	11	10	12
		R	6.33	5.88	6.56	7.42	8.81	7.57	7.91	8.53	8.67	7.38	8.88	9.90	6.43	8.44	10.30	9.46	11.60	9.17	8.88	9.24
		F_{IS}	0.05	0.01	-0.08	-0.07	-0.06	0.20	0.02	0.19	-0.02	0.00	-0.09	0.00	0.03	0.00	0.07	-0.05	0.09	-0.08	-0.01	0.09
		H_E	0.53	0.72	0.73	0.80	0.83	0.74	0.81	0.83	0.74	0.82	0.77	0.82	0.68	0.82	0.86	0.84	0.89	0.85	0.85	0.84
		H_O	0.50	0.71	0.78	0.85	0.88	0.59	0.80	0.68	0.75	0.82	0.84	0.82	0.66	0.82	0.80	0.89	0.81	0.92	0.85	0.77
Aa081	9	N	53	64	37	56	36	48	31	34	32	33	25	38	54	55	39	55	51	50	48	47
Range	145-173	N_a	7	5	5	5	6	6	5	4	5	5	5	5	5	5	7	5	6	5	5	5
		R	5.35	4.84	4.64	4.90	5.33	5.44	5.00	4.00	4.75	4.73	5.00	4.86	4.74	4.68	6.22	4.90	5.41	4.46	4.50	4.88
		F_{IS}	-0.04	-0.01	0.16	0.08	0.16	0.09	0.20	0.14	-0.12	0.12	0.06	0.07	0.03	0.13	-0.05	0.04	-0.07	-0.03	-0.18	0.06
		H_E	0.71	0.73	0.71	0.76	0.73	0.73	0.76	0.74	0.75	0.75	0.76	0.71	0.67	0.73	0.78	0.72	0.68	0.68	0.71	0.70
		H_O	0.74	0.73	0.59	0.70	0.61	0.67	0.61	0.65	0.84	0.67	0.72	0.66	0.65	0.64	0.82	0.69	0.73	0.70	0.83	0.66
Aa070	11	N	53	67	40	56	36	48	31	36	34	33	25	37	54	47	40	55	48	48	49	45
Range	210-282	N_a	7	5	3	7	7	5	6	9	8	6	7	7	6	7	8	6	8	10	6	8

		R	4.99	4.11	2.59	5.59	6.75	4.26	5.72	7.85	7.53	5.65	6.84	6.71	4.84	6.40	6.96	5.58	6.72	7.64	5.44	7.00
		F_{IS}	0.08	-0.02	-0.06	-0.01	0.03	-0.12	-0.09	0.01	-0.01	-0.06	0.11	0.08	0.10	-0.10	-0.12	0.10	0.06	0.09	0.02	-0.08
		H_E	0.24	0.29	0.14	0.46	0.60	0.30	0.50	0.67	0.73	0.57	0.63	0.61	0.53	0.68	0.69	0.69	0.75	0.62	0.67	0.66
		H_O	0.23	0.30	0.15	0.46	0.58	0.33	0.55	0.67	0.74	0.61	0.56	0.57	0.48	0.74	0.78	0.62	0.71	0.56	0.65	0.71
Aa091	6	N	57	35	42	60	46	49	44	36	34	31	25	38	53	57	41	57	58	51	45	47
Range	233-253	N_a	5	6	5	5	4	4	5	5	5	6	6	5	5	6	6	5	6	5	6	5
		R	4.97	5.68	4.91	4.66	3.52	3.49	4.34	4.99	4.91	5.72	5.96	4.98	4.84	5.32	5.70	4.56	5.01	4.45	5.42	4.40
		F_{IS}	-0.08	0.13	-0.09	0.08	0.13	-0.20	-0.20	0.04	0.06	0.16	-0.14	0.20	-0.05	-0.08	-0.07	0.24	0.00	0.00	0.07	0.22
		H_E	0.68	0.69	0.68	0.64	0.65	0.61	0.63	0.70	0.68	0.62	0.74	0.69	0.72	0.70	0.69	0.64	0.62	0.61	0.67	0.57
		H_O	0.74	0.60	0.74	0.58	0.57	0.73	0.75	0.67	0.65	0.52	0.84	0.55	0.75	0.75	0.73	0.49	0.62	0.61	0.62	0.45
Aa093	9	N	54	67	33	60	41	47	38	33	32	32	25	38	55	49	40	58	59	49	39	49
Range	181-245	N_a	3	5	3	4	3	3	4	5	3	3	3	4	4	4	7	6	7	5	5	6
		R	2.83	3.65	2.98	3.19	2.99	2.99	3.69	4.64	2.99	2.97	2.96	3.74	3.43	3.47	5.86	3.86	4.55	3.97	4.33	4.82
		F_{IS}	-0.01	-0.15	0.23	0.09	-0.06	0.23	-0.05	0.20	-0.13	-0.06	-0.10	0.07	0.11	-0.04	0.16	-0.06	0.20	0.00	0.04	0.13
		H_E	0.52	0.56	0.55	0.46	0.30	0.38	0.18	0.30	0.28	0.18	0.22	0.28	0.30	0.28	0.35	0.18	0.28	0.43	0.43	0.45
		H_O	0.52	0.64	0.42	0.42	0.32	0.30	0.18	0.24	0.31	0.19	0.24	0.26	0.27	0.29	0.30	0.19	0.22	0.43	0.41	0.39
Ap033	10	N	54	68	43	63	42	48	38	33	33	32	25	38	57	48	42	57	59	52	40	49
Range	203-245	N_a	5	5	7	6	5	5	5	6	5	6	5	5	7	7	6	5	6	5	5	5
		R	4.99	4.99	6.11	5.77	4.99	4.97	5.00	5.45	4.93	5.93	4.96	5.00	5.81	6.24	5.54	4.99	5.40	4.99	4.94	4.97
		F_{IS}	0.04	-0.12	0.04	0.00	-0.06	-0.01	0.02	-0.12	0.00	-0.07	0.15	0.08	-0.09	0.19	-0.18	0.06	-0.04	-0.06	0.07	0.01
		H_E	0.73	0.73	0.73	0.73	0.65	0.64	0.73	0.71	0.73	0.73	0.70	0.74	0.74	0.74	0.73	0.73	0.75	0.74	0.72	0.76
		H_O	0.70	0.82	0.70	0.73	0.69	0.65	0.71	0.79	0.73	0.78	0.60	0.68	0.81	0.60	0.86	0.68	0.78	0.79	0.68	0.76
All loci		R	4.31	4.21	4.00	4.63	4.58	4.35	4.60	5.05	4.82	4.78	5.04	5.24	4.31	5.01	5.49	5.10	5.49	4.90	4.97	5.07
		F_{IS}	-0.02	-0.03	0.02	0.00	0.02	0.08	0.01	0.09	-0.03	0.04	0.02	0.10	-0.02	0.02	-0.04	0.07	0.05	-0.01	0.03	0.05
		H_E	0.51	0.56	0.56	0.59	0.56	0.54	0.56	0.60	0.61	0.61	0.60	0.61	0.58	0.61	0.64	0.62	0.64	0.62	0.63	0.63
		H_O	0.54	0.58	0.55	0.59	0.54	0.50	0.56	0.55	0.63	0.58	0.59	0.55	0.59	0.60	0.67	0.57	0.60	0.63	0.62	0.60

Table S2: Blueback herring genetic diversity statistics: number of specimens genotyped (N), number of alleles per locus (N_a), allelic richness (R standardized to N=26), observed heterozygosity (H_O), expected heterozygosity (H_E), and inbreeding coefficient (F_{IS}).

			Population																			
**Locus**	**A**		EMA	STG	EXE	MYS	MON	GIL	CON	HUD	DEL	NAN	JAM	RAP	CHO	ROA	NEU	CFE	SAN	ALT	SAV	STJ
Ap037	28	N	55	41	41	56	40	33	136	75	48	24	90	46	60	50	58	55	61	51	50	37
Range	205-364	N_a	7	6	6	9	4	7	13	10	11	4	15	11	9	9	11	5	6	5	7	5
		R	6.05	5.46	5.50	6.49	3.53	6.32	8.14	5.78	8.41	7.63	8.08	7.72	7.11	6.60	8.09	4.47	5.35	4.40	5.33	4.26
		F_{IS}	0.26	-0.03	0.18	0.05	0.04	-0.11	0.15	0.14	0.13	0.06	0.08	0.13	0.19	-0.10	0.25	0.31	0.09	0.32	0.13	0.08
		H_E	0.64	0.59	0.56	0.45	0.29	0.44	0.45	0.28	0.43	0.26	0.43	0.35	0.37	0.38	0.48	0.58	0.63	0.52	0.51	0.51
		H_O	0.47	0.61	0.46	0.43	0.28	0.48	0.38	0.24	0.38	0.25	0.40	0.30	0.30	0.42	0.36	0.40	0.57	0.35	0.44	0.35
Aa046	9	N	53	41	41	66	46	38	142	76	48	24	94	56	69	50	62	56	60	52	51	37
Range	212-264	N_a	4	4	5	7	4	6	6	6	7	6	6	7	6	5	6	6	3	4	4	5
		R	4.00	3.63	4.61	5.55	3.78	4.93	4.71	5.05	6.18	5.56	5.26	6.06	5.36	4.77	4.73	5.29	3.00	3.74	3.51	3.57
		F_{IS}	-0.17	-0.14	-0.12	0.13	0.24	-0.06	0.03	-0.02	0.08	0.01	-0.06	-0.04	0.16	-0.01	0.00	0.03	0.02	0.08	0.01	-0.04
		H_E	0.58	0.41	0.33	0.57	0.26	0.20	0.39	0.35	0.38	0.46	0.45	0.46	0.40	0.48	0.42	0.48	0.44	0.46	0.49	0.54
		H_O	0.68	0.46	0.37	0.50	0.20	0.21	0.38	0.36	0.35	0.50	0.48	0.48	0.33	0.48	0.42	0.46	0.43	0.42	0.49	0.57
Aa074	19	N	54	41	40	51	35	30	135	74	35	24	89	51	65	46	61	53	60	52	51	37
Range	210-262	N_a	6	5	7	7	7	5	8	10	8	7	11	8	10	10	8	8	10	8	6	5
		R	5.35	4.98	6.49	5.73	6.42	4.85	6.47	7.72	7.80	7.62	7.78	6.75	8.38	8.64	7.33	6.97	7.92	7.12	5.51	6.68
		F_{IS}	0.14	-0.03	-0.29	0.06	0.09	0.14	0.02	0.14	0.13	-0.02	-0.03	-0.06	-0.03	0.06	0.19	0.17	0.06	0.11	0.08	0.10
		H_E	0.71	0.76	0.70	0.69	0.72	0.70	0.75	0.74	0.75	0.70	0.71	0.70	0.74	0.76	0.79	0.77	0.78	0.78	0.74	0.60
		H_O	0.61	0.78	0.90	0.65	0.66	0.60	0.73	0.64	0.66	0.67	0.73	0.75	0.77	0.72	0.64	0.64	0.73	0.69	0.69	0.51
Aa082	21	N	57	41	41	45	42	38	131	71	47	24	84	55	55	44	62	55	56	51	50	37
Range	156-268	N_a	6	8	10	11	8	8	15	13	14	8	10	10	10	11	15	11	9	9	10	8
		R	4.98	7.07	8.57	8.54	7.03	7.58	10.02	7.79	11.55	8.21	7.43	8.92	8.34	9.74	11.26	8.18	7.55	7.66	9.22	7.27
		F_{IS}	0.10	0.11	-0.01	0.26	-0.11	-0.12	0.17	0.17	-0.04	0.05	0.16	0.17	0.13	0.09	0.32	0.14	0.12	0.16	0.27	0.07
		H_E	0.59	0.68	0.70	0.60	0.75	0.82	0.75	0.68	0.80	0.70	0.69	0.72	0.69	0.75	0.75	0.62	0.73	0.77	0.79	0.61
		H_O	0.53	0.61	0.71	0.44	0.83	0.92	0.63	0.56	0.83	0.67	0.58	0.60	0.60	0.68	0.52	0.53	0.64	0.65	0.58	0.54
Ap071	20	N	52	42	39	56	50	37	135	53	39	24	83	44	67	46	62	57	40	47	37	35
Range	165-283	N_a	10	9	10	8	7	7	11	11	11	10	14	10	11	9	11	9	9	8	8	7
		R	8.95	8.61	9.19	7.63	6.29	6.67	9.18	9.62	9.93	8.80	10.25	9.17	9.10	8.38	9.43	8.30	8.30	7.89	7.59	7.91
		F_{IS}	0.06	-0.02	0.06	0.08	0.04	0.02	0.09	0.00	0.10	-0.07	-0.03	0.02	0.13	0.04	-0.05	0.03	0.08	0.02	0.02	-0.05
		H_E	0.86	0.86	0.84	0.83	0.77	0.74	0.86	0.83	0.85	0.80	0.86	0.84	0.85	0.86	0.86	0.87	0.85	0.85	0.80	0.83
		H_O	0.81	0.88	0.79	0.77	0.74	0.73	0.78	0.83	0.77	0.88	0.88	0.82	0.75	0.83	0.90	0.84	0.78	0.83	0.78	1.00
Ap038	10	N	57	37	40	46	45	35	130	60	35	21	92	58	69	48	62	56	60	52	50	37
Range	210-250	N_a	3	3	2	5	4	3	5	5	6	3	5	3	5	4	3	3	3	3	2	2
		R	2.70	2.70	2.00	4.34	2.73	2.73	3.69	3.80	5.36	3.42	3.57	2.83	3.74	3.08	2.81	2.46	2.68	2.50	2.00	2.00
		F_{IS}	-0.08	-0.06	-0.13	0.16	0.33	-0.03	0.00	0.00	0.51	-0.08	0.12	0.12	0.19	0.05	0.03	0.04	0.07	0.00	0.23	0.00
		H_E	0.18	0.15	0.22	0.26	0.07	0.11	0.30	0.22	0.29	0.22	0.21	0.18	0.21	0.24	0.23	0.22	0.38	0.38	0.29	0.28
		H_O	0.19	0.16	0.25	0.22	0.04	0.11	0.30	0.22	0.14	0.24	0.18	0.16	0.17	0.23	0.23	0.21	0.35	0.38	0.22	0.32
Ap010	8	N	57	42	41	53	48	35	139	72	48	24	97	58	66	50	64	57	60	52	50	37
Range	221-241	N_a	3	3	2	3	3	3	3	3	4	4	3	4	6	5	3	4	4	4	4	3
		R	2.69	2.86	1.95	2.68	2.96	2.94	2.87	2.59	3.45	5.26	2.94	3.15	4.64	4.04	2.99	3.37	3.81	3.63	3.88	3.00
		F_{IS}	0.31	-0.07	-0.03	-0.04	0.11	0.19	-0.06	-0.08	0.15	0.07	0.02	0.11	0.10	0.04	0.03	-0.15	-0.12	-0.07	0.08	-0.09
		H_E	0.10	0.18	0.07	0.11	0.21	0.21	0.21	0.17	0.29	0.33	0.36	0.27	0.45	0.37	0.35	0.41	0.39	0.38	0.30	0.46
		H_O	0.07	0.19	0.07	0.11	0.19	0.17	0.22	0.18	0.25	0.38	0.35	0.24	0.41	0.36	0.34	0.47	0.43	0.40	0.28	0.49
Ap047	7	N	55	42	40	51	46	38	138	48	35	24	88	39	68	49	61	57	49	36	48	37
Range	212-250	N_a	3	4	3	3	3	3	3	3	6	3	3	3	3	3	3	3	3	3	3	3

		R	3.00	3.62	3.00	3.00	3.00	3.00	3.00	3.00	5.62	3.00	3.00	3.00	3.00	3.00	3.00	3.00	3.00	3.00	3.00	3.00
		F_{IS}	-0.08	-0.15	0.09	0.12	-0.13	0.07	0.18	0.09	0.20	-0.06	0.13	0.33	-0.04	-0.02	0.15	0.01	0.07	-0.11	-0.03	0.05
		H_E	0.59	0.60	0.66	0.62	0.58	0.59	0.60	0.55	0.60	0.52	0.52	0.50	0.58	0.46	0.56	0.55	0.59	0.48	0.47	0.40
		H_O	0.64	0.69	0.60	0.55	0.65	0.55	0.49	0.50	0.49	0.63	0.45	0.33	0.60	0.47	0.48	0.54	0.55	0.53	0.48	0.41
Aa070	9	N	57	41	40	55	46	33	140	45	42	24	90	49	66	49	62	57	51	51	49	36
Range	246-282	N_a	4	3	3	3	5	2	5	3	6	3	5	3	5	3	3	2	3	3	4	3
		R	3.44	2.87	3.00	2.47	3.94	2.00	3.59	3.00	4.95	2.92	4.08	2.96	3.66	2.90	2.81	2.00	2.27	2.51	3.53	2.75
		F_{IS}	0.00	-0.05	0.07	0.19	-0.03	0.15	0.05	0.15	0.31	0.05	0.05	0.25	-0.10	0.19	-0.16	-0.12	-0.01	-0.14	0.03	-0.06
		H_E	0.44	0.44	0.46	0.36	0.32	0.32	0.41	0.49	0.52	0.50	0.48	0.46	0.39	0.43	0.36	0.20	0.06	0.24	0.34	0.29
		H_O	0.44	0.46	0.43	0.29	0.33	0.27	0.39	0.42	0.36	0.50	0.46	0.35	0.42	0.35	0.42	0.23	0.06	0.27	0.33	0.28
Ap070	18	N	47	40	40	46	49	38	140	72	43	24	93	48	66	49	65	57	60	52	50	37
Range	162-253	N_a	11	9	8	7	6	5	6	8	7	6	6	8	6	6	7	6	6	6	6	5
		R	9.99	8.49	6.94	6.35	5.95	4.99	5.98	6.21	6.82	5.98	5.73	6.87	5.61	5.88	6.54	5.76	5.82	5.98	5.41	5.99
		F_{IS}	0.33	0.39	0.09	0.02	-0.01	-0.02	0.05	0.00	-0.01	-0.03	-0.01	-0.09	-0.07	-0.07	0.07	-0.07	-0.16	0.06	-0.04	-0.06
		H_E	0.85	0.82	0.74	0.75	0.73	0.67	0.80	0.77	0.79	0.77	0.78	0.77	0.76	0.78	0.79	0.75	0.79	0.80	0.77	0.74
		H_O	0.57	0.50	0.68	0.74	0.73	0.68	0.76	0.76	0.79	0.75	0.78	0.83	0.82	0.84	0.74	0.81	0.92	0.75	0.80	0.81
Aa091	7	N	56	26	40	50	49	34	139	75	45	24	96	57	67	49	65	57	58	45	50	37
Range	233-257	N_a	5	4	5	5	5	4	6	5	5	6	7	7	5	5	5	5	5	5	5	3
		R	4.42	4.00	4.53	4.47	4.98	3.98	4.83	4.70	4.87	5.17	5.20	5.73	4.62	4.77	4.91	3.91	4.69	4.98	4.99	3.23
		F_{IS}	0.10	0.16	-0.15	-0.10	-0.01	0.11	0.05	-0.03	-0.15	-0.01	-0.06	-0.07	0.06	0.06	0.04	-0.02	0.03	0.16	0.07	-0.03
		H_E	0.50	0.55	0.48	0.66	0.69	0.56	0.57	0.58	0.47	0.45	0.54	0.54	0.57	0.52	0.56	0.57	0.64	0.66	0.68	0.43
		H_O	0.45	0.46	0.55	0.72	0.69	0.50	0.54	0.60	0.53	0.50	0.57	0.58	0.54	0.49	0.54	0.58	0.62	0.56	0.64	0.43
Aa093	13	N	47	38	35	53	43	36	134	53	46	21	84	55	65	47	65	56	57	49	44	37
Range	177-251	N_a	7	9	6	7	6	5	7	8	7	5	7	8	6	6	6	5	8	8	6	7
		R	5.94	8.26	5.74	6.68	5.21	5.00	6.13	7.15	6.63	5.11	5.44	6.36	4.91	5.87	5.64	4.42	6.43	6.52	5.50	5.66
		F_{IS}	0.04	0.27	0.04	0.18	-0.04	0.08	0.09	0.13	-0.01	-0.16	-0.11	0.11	0.00	-0.14	-0.12	0.12	0.20	0.00	0.16	0.14
		H_E	0.71	0.79	0.71	0.69	0.72	0.75	0.73	0.71	0.65	0.62	0.63	0.67	0.61	0.66	0.65	0.63	0.61	0.66	0.60	0.43
		H_O	0.68	0.58	0.69	0.57	0.74	0.69	0.66	0.62	0.65	0.67	0.70	0.60	0.62	0.74	0.72	0.55	0.49	0.65	0.50	0.32
Ap033	10	N	54	38	41	55	48	37	138	51	41	24	90	55	65	48	64	57	61	52	51	37
Range	197-225	N_a	6	6	7	6	4	6	8	7	7	7	8	7	7	8	8	8	6	7	7	7
		R	5.43	5.68	6.44	5.05	3.91	5.40	6.56	6.39	6.97	6.65	6.52	6.20	6.60	7.62	6.84	7.02	5.92	6.76	6.49	6.28
		F_{IS}	0.08	-0.14	-0.11	0.05	0.36	0.10	0.05	0.04	0.20	-0.04	0.03	-0.01	-0.09	0.15	0.08	0.19	0.37	0.13	0.19	0.42
		H_E	0.54	0.65	0.59	0.59	0.55	0.63	0.68	0.69	0.70	0.75	0.68	0.74	0.72	0.68	0.70	0.56	0.67	0.66	0.68	0.52
		H_O	0.50	0.74	0.66	0.56	0.35	0.57	0.64	0.67	0.56	0.79	0.66	0.75	0.78	0.58	0.64	0.46	0.43	0.58	0.55	0.43
All loci		R	5.15	5.25	5.23	5.31	4.59	4.64	5.78	5.60	6.81	5.79	5.79	5.82	5.77	5.79	5.87	5.01	5.13	5.13	5.07	4.74
		F_{IS}	0.09	0.05	-0.01	0.09	0.03	0.04	0.08	0.07	0.10	-0.02	0.02	0.06	0.03	0.03	0.08	0.07	0.07	0.07	0.09	0.05
		H_E	0.56	0.58	0.54	0.55	0.51	0.52	0.58	0.54	0.58	0.55	0.56	0.55	0.57	0.57	0.58	0.56	0.58	0.59	0.57	0.51
		H_O	0.51	0.55	0.55	0.50	0.50	0.50	0.53	0.51	0.52	0.57	0.56	0.52	0.55	0.55	0.53	0.52	0.54	0.54	0.52	0.50

Table S3: Alewife genetic differentiation. Pairwise F_{ST} values (θ; Weir and Cockerham 1984) below diagonal (non-significant values in bold) and standardized F_{ST} values (F'_{ST}; Hedrick 2005) above diagonal.

	EMA	STG	LAM	MYS	MON	TOW	GIL	THA	BRI	CON	QUI	HOU	MIA	HUD	DEL	NAN	RAP	CHO	ROA	ALL
EMA	.	0.041	0.040	0.143	0.218	0.212	0.214	0.205	0.201	0.241	0.202	0.170	0.220	0.182	0.240	0.287	0.328	0.321	0.352	0.336
STG	**0.019**	.	-0.003	0.052	0.157	0.141	0.168	0.140	0.154	0.181	0.150	0.099	0.130	0.146	0.176	0.212	0.232	0.210	0.240	0.216
LAM	**0.019**	**-0.002**	.	0.074	0.175	0.157	0.172	0.160	0.171	0.185	0.172	0.120	0.148	0.167	0.197	0.219	0.238	0.210	0.254	0.227
MYS	0.063	0.022	0.031	.	0.070	0.067	0.051	0.033	0.077	0.060	0.061	0.014	0.045	0.060	0.066	0.115	0.105	0.101	0.119	0.100
MON	0.101	0.070	0.078	0.030	.	0.037	0.021	0.014	0.084	0.089	0.031	0.045	0.085	0.091	0.111	0.153	0.159	0.201	0.202	0.208
TOW	0.099	0.064	0.071	0.029	**0.017**	.	0.048	0.054	0.118	0.099	0.068	0.065	0.095	0.129	0.147	0.201	0.200	0.218	0.207	0.214
GIL	0.097	0.074	0.076	**0.021**	**0.009**	0.022	.	0.008	0.051	0.051	0.033	0.042	0.055	0.071	0.089	0.144	0.147	0.170	0.184	0.166
THA	0.090	0.059	0.068	**0.013**	**0.006**	0.023	**0.004**	.	0.016	0.023	0.001	0.002	0.036	0.025	0.026	0.081	0.090	0.128	0.130	0.127
BRI	0.087	0.065	0.071	0.031	0.035	0.051	**0.021**	**0.006**	.	0.071	0.013	0.042	0.069	0.052	0.049	0.104	0.133	0.175	0.189	0.170
CON	0.105	0.076	0.077	0.024	0.038	0.043	0.021	**0.009**	0.028	.	0.061	0.028	0.087	0.030	0.030	0.055	0.057	0.083	0.100	0.087
QUI	0.089	0.064	0.073	0.024	**0.013**	0.030	**0.014**	**0.001**	**0.005**	**0.024**	.	0.018	0.057	0.032	0.038	0.087	0.129	0.165	0.153	0.158
HOU	0.074	0.041	0.050	**0.006**	**0.019**	0.028	**0.017**	**0.001**	**0.016**	**0.011**	**0.007**	.	0.028	0.014	0.018	0.052	0.057	0.106	0.102	0.100
MIA	0.098	0.056	0.064	0.019	0.037	0.042	0.024	0.015	0.028	0.035	0.023	**0.011**	.	0.074	0.080	0.111	0.119	0.122	0.143	0.105
HUD	0.078	0.060	0.069	0.024	0.037	0.055	0.029	**0.010**	0.020	**0.012**	**0.013**	**0.006**	0.030	.	0.009	0.048	0.077	0.125	0.130	0.114
DEL	0.100	0.071	0.079	0.025	0.044	0.060	0.035	**0.010**	0.018	0.011	**0.014**	**0.007**	0.031	**0.004**	.	0.011	0.027	0.070	0.059	0.069
NAN	0.123	0.088	0.091	0.046	0.063	0.085	0.059	0.032	0.040	0.021	0.034	0.020	0.045	0.018	**0.004**	.	0.023	0.054	0.054	0.065
RAP	0.138	0.095	0.096	0.041	0.065	0.083	0.059	0.034	0.050	0.022	0.049	0.022	0.047	0.029	**0.010**	0.009	.	0.041	0.042	0.059
CHO	0.137	0.087	0.086	0.040	0.083	0.092	0.069	0.050	0.067	0.032	0.064	0.041	0.049	0.048	0.026	0.021	0.015	.	-0.001	-0.001
ROA	0.148	0.097	0.102	0.046	0.081	0.085	0.073	0.050	0.071	0.038	0.058	0.039	0.056	0.049	**0.021**	0.020	0.016	**-0.001**	.	0.009
ALL	0.143	0.089	0.092	0.039	0.085	0.089	0.066	0.049	0.065	0.033	0.061	0.038	0.042	0.043	0.025	0.025	0.022	**-0.001**	**0.003**	.

Table S4: Blueback herring genetic differentiation. Pairwise F_{ST} values (θ; Weir and Cockerham 1984) below diagonal (non-significant values in bold) and standardized F_{ST} values (F'_{ST}; Hedrick 2005) above diagonal.

	EMA	STG	EXE	MYS	MON	GIL	CON	HUD	DEL	NAN	JAM	RAP	CHO	ROA	NEU	CFE	SAN	ALT	SAV	STJ
EMA	.	0.025	0.054	0.086	0.105	0.103	0.030	0.065	0.053	0.062	0.065	0.070	0.073	0.067	0.062	0.087	0.114	0.124	0.110	0.175
STG	**0.011**	.	0.067	0.114	0.105	0.102	0.054	0.090	0.092	0.104	0.112	0.107	0.098	0.117	0.100	0.134	0.149	0.156	0.148	0.233
EXE	0.024	0.029	.	0.096	0.078	0.099	0.044	0.090	0.080	0.089	0.088	0.106	0.092	0.093	0.075	0.112	0.125	0.133	0.146	0.197
MYS	0.038	0.050	0.043	.	0.073	0.059	0.058	0.074	0.115	0.110	0.099	0.104	0.095	0.114	0.104	0.128	0.122	0.120	0.101	0.204
MON	0.049	0.048	0.037	0.034	.	0.015	0.057	0.083	0.096	0.098	0.082	0.098	0.079	0.096	0.084	0.109	0.131	0.130	0.124	0.203
GIL	0.047	0.046	0.047	0.027	**0.007**	.	0.047	0.055	0.076	0.078	0.066	0.070	0.064	0.084	0.073	0.103	0.125	0.119	0.111	0.218
CON	0.013	0.023	0.019	0.025	0.026	0.021	.	0.016	0.015	0.018	0.027	0.024	0.027	0.029	0.022	0.066	0.083	0.080	0.071	0.175
HUD	0.029	0.040	0.041	0.034	0.039	0.026	0.007	.	0.009	0.013	0.015	0.002	0.019	0.015	0.018	0.066	0.108	0.079	0.059	0.186
DEL	0.023	0.039	0.035	0.050	0.044	0.034	**0.006**	**0.004**	.	-0.008	0.005	-0.003	0.014	-0.008	0.002	0.058	0.092	0.068	0.061	0.167
NAN	0.028	0.045	0.041	0.050	0.046	0.037	**0.008**	**0.006**	**-0.003**	.	0.001	-0.007	0.009	0.002	0.011	0.083	0.115	0.096	0.080	0.184
JAM	0.029	0.048	0.039	0.044	0.038	0.030	0.012	**0.007**	**0.002**	**0.001**	.	0.001	0.007	-0.005	0.000	0.033	0.078	0.061	0.055	0.147
RAP	0.031	0.046	0.048	0.046	0.046	0.032	**0.011**	**0.001**	**-0.001**	**-0.003**	**0.000**	.	0.007	-0.002	0.004	0.060	0.099	0.077	0.060	0.165
CHO	0.032	0.042	0.041	0.042	0.036	0.029	0.011	**0.008**	**0.006**	**0.004**	**0.003**	**0.003**	.	0.011	0.004	0.031	0.063	0.059	0.049	0.126
ROA	0.029	0.050	0.041	0.050	0.044	0.038	0.012	**0.007**	**-0.003**	**0.001**	**-0.002**	**-0.001**	**0.005**	.	-0.007	0.033	0.075	0.047	0.049	0.140
NEU	0.027	0.042	0.033	0.045	0.038	0.033	0.010	0.008	**0.001**	**0.005**	**0.000**	**0.002**	**0.002**	**-0.003**	.	0.018	0.052	0.037	0.042	0.131
CFE	0.038	0.059	0.051	0.057	0.051	0.048	0.029	0.030	0.025	0.037	0.014	0.027	0.014	0.015	0.008	.	0.022	0.027	0.036	0.093
SAN	0.049	0.063	0.054	0.053	0.059	0.056	0.035	0.047	0.039	0.050	0.034	0.043	0.027	0.032	0.022	0.010	.	0.007	0.023	0.097
ALT	0.053	0.065	0.058	0.051	0.058	0.053	0.034	0.034	0.028	0.042	0.026	0.033	0.025	0.020	0.016	0.012	**0.003**	.	0.007	0.103
SAV	0.048	0.063	0.064	0.044	0.057	0.050	0.030	0.026	0.026	0.035	0.024	0.026	0.021	0.021	0.018	0.016	0.010	**0.003**	.	0.087
STJ	0.081	0.106	0.093	0.095	0.099	0.106	0.079	0.088	0.076	0.087	0.067	0.077	0.058	0.064	0.059	0.043	0.044	0.046	0.040	.

Table S5: AMOVA results. Clusters refer to genetic stocks identified using STRUCTURE v. 2.3.3 (Pritchard *et al.* 2000; Falush *et al.* 2003) and BAPS v. 5.3 (Corander *et al.* 2006).

**Alewife**					
**Variance component**	**d.f.**	**Sum of squares**	**% total variance**	**F-statistic**	**P**
Among rivers	7	59.60	1.65	0.017	<0.001
Among collections within rivers	8	23.58	-0.05	0.000	0.556
Among individuals within collections	872	2632.41	98.40	0.016	0.044
Among clusters	2	186.55	4.70	0.047	<0.001
Among rivers within clusters	17	113.40	1.30	0.014	<0.001
Among individuals within rivers	1940	5549.55	94.00	0.060	<0.001
**Blueback herring**					
**Variance component**	**d.f.**	**Sum of squares**	**% total variance**	**F-statistic**	**P**
Among rivers	4	79.96	3.21	0.032	0.015
Among collections within rivers	6	20.11	0.17	0.002	0.165
Among individuals within collections	841	2529.65	96.63	0.034	<0.001
Among clusters	3	144.85	2.45	0.024	<0.001
Among rivers within clusters	16	103.39	0.82	0.008	<0.001
Among individuals within rivers	2480	7786.68	96.74	0.033	<0.001

Table S6: Alewife demographic time series results with genetic stock assignments listed for each river (NNE-Northern New England, SNE-Southern New England, MAT-Mid-Atlantic). Non-parametric linear regression slopes are given (significant values in bold).

			**Mean Length**		**Run Size**	
**River**	**State**	**Genetic Stock**	**Females**	**Males**	**Counts**	**CPUE**
Union	ME	NNE			0.013	
Damariscotta	ME	NNE			-0.005	
Androscoggin	ME	NNE	-0.150	-0.234	0.027	
Coheco	NH	NNE	0.000	-0.091		
Exeter	NH	NNE	-0.500	-0.583		
Lamprey	NH	NNE	0.127	-0.038		
Winnicut	NH	NNE	0.774	0.050		
Parker	MA	SNE			**-0.041**	
Stony Brook	MA	SNE	**-1.600***			
Monument	MA	SNE	**-1.048**	**-0.873**	-0.006	
Mattipoisett	MA	SNE			-0.053	
Nemasket	MA	SNE			-0.063	
Nonquit	RI	SNE			**-0.156**	
Buckeye Brook	RI	SNE			-0.014	
Gilbert Stuart	RI	SNE			0.009	
Shetucket	CT	SNE			-0.022	
Bride Brook	CT	SNE			0.047	
Mill Brook	CT	SNE			-0.153	
Farmington	CT	SNE			-0.198	
Naugatuck	CT	SNE			-0.409	
Mianus	CT	SNE			0.139	
Hudson	NY	SNE	**-1.333**	**-1.261**		
Nanticoke	MD	MAT	0.174	0.000		
Rappahannock	VA	MAT				0.009
York	VA	MAT				**0.099**
James	VA	MAT				-0.048
Chowan	NC	MAT	**-0.620**	**-0.600**	**-0.076**	**-0.080**

* Females and males combined in time series.

Table S7: Blueback herring demographic time series results with genetic stock assignments listed for each river (NNE-Northern New England, SNE-Southern New England, MAT-Mid-Atlantic, SAT-South Atlantic). Non-parametric linear regression slopes are given (significant values in bold).

			Mean Length		Run Size	
**River**	**State**	**Genetic Stock**	**Females**	**Males**	**Counts**	**CPUE**
Cocheco	NH	NNE	-0.160	-0.696		
Oyster	NH	NNE	**-0.700**	**-1.000**		
Winnicut	NH	NNE	-0.417	-0.083		
Monument	MA	SNE	**-1.056**	**-0.888**	**-0.060**	
Shetucket	CT	MAT			**-0.013**	
Connecticut	CT	MAT			0.000	
Farmington	CT	MAT			-0.198	
Naugatuck	CT	MAT			0.000	
Mianus	CT	MAT			0.156	
Hudson	NY	MAT	**-1.500**	**-1.167**		
Nanticoke	MD	MAT	**-0.916**	**-0.879**		
Chowan	NC	MAT	**-0.636**	**-0.697**	**-0.065**	**-0.051**
Santee	SC	SAT	**-0.636**	**-0.583**	0.199	0.049
Cooper	SC	SAT				**-0.034**
St Johns	FL	SAT	**-0.651**	-0.711		

Table S8: Organizations and individuals that provided assistance with sample collection.

Organization	Samplers
University of Southern Maine	K. Wilson, E. M. Labbe
Gulf of Maine Research Institute	J. Stockwell, Z. Whitener
New Hampshire Fish and Game Department	K. Sullivan
Massachusetts Department of Marine Fisheries	J. Sheppard, S. Elzey, B. Gahagan
Rhode Island Department of Fish and Wildlife	P. Edwards
Connecticut Department of Environmental Protection	D. Ellis
University of Connecticut	J. Vokoun
New York State Department of Environmental Conservation	R. Adams, W. Eakin
State University of New York - ESF	S. M. Turner
New Jersey Department of Fish and Wildlife	H. Corbett
Delaware Department of Fish and Wildlife	M.Stangl
Maryland Department of Natural Resources	C. Stence, T. Jarzynski, E. Durell, K. Capossela
Virginia Department of Game and Inland Fisheries	E. Brittle, A. Weaver
Virginia Institute of Marine Science	L. Machut
North Carolina Division of Marine Fisheries	A. Kenyon
North Carolina State University	P. McClellen-Green, J. Hightower, J. Hughes
North Carolina Wildlife Resources Commission	J. McCargo, R. Barwick, K. Ashley, T. Rachels, B. Ricks, K. Rundle, K. Dockendorf, J. Homan, B. Wynne
South Carolina Department of Natural Resources	B. Post, B. Eleby
Georgia Department of Natural Resources	D. Harrison, B. Ballard,T. Mathes
Florida Fish and Wildlife Commission	R. Hyle
University of Florida	D. Dutterer
US Fish and Wildlife Service	K. Gustafson, K. Sprankle, J. McKeon, M. Odom, K. Ware, J. Henne
National Marine Fisheries Service	M. Scripter, S. Rowe
